# Discordant Interleukin-27 (IL-27) Subunit Transcription in Human Sepsis Exposes a Molecular-Attribution Gap

**DOI:** 10.3390/biomedicines14092035

**Published:** 2026-09-10

**Authors:** Yue Zhang, Qi-Shun Sun

**Affiliations:** 1 Department of Anesthesiology, Shanghai Geriatric Medical Center, Shanghai 201104, China; zhangyuee012@163.com; 2 Department of Anesthesiology, Zhongshan Hospital, Fudan University, Shanghai 200032, China

**Keywords:** sepsis, interleukin-27, EBI3, monocyte heterogeneity, single-cell RNA sequencing, subunit co-detection

## Abstract

**Background/Objectives**: Interleukin-27 (IL-27) is an obligate EBI3–p28 heterodimer, yet sepsis studies commonly measure and target the axis as a single entity. We tested whether both subunit transcripts are coordinately induced in septic monocytes and whether the axis has functional consequences. **Methods**: We integrated 272,993 whole-blood single-cell transcriptomes from 39 individuals, six additional single-cell cohorts and eight bulk cohorts, with two human macrophage datasets, a tolerant-like THP-1 model and caecal ligation and puncture in male C57BL/6 mice. **Results**: Across three cohorts (166 donor records), *EBI3* ranked 137th of 13,872 genes whereas *IL-27* ranked 9413th. Against post-cardiac-surgery controls, detection odds ratios (ORs) were 11.0 for *EBI3* and 0.78 for *IL-27* (ratio 13.3). Thirteen of 7164 septic monocytes carried both transcripts, matching the chance expectation (co-detection odds ratio 0.91, 95% CI 0.48–1.75) against 5.6–346.7 for concordantly transcribed benchmark complexes; the receiver-side module estimate was β = −0.0151. The imbalance was reproduced in human macrophages. In tolerant-like THP-1 cells, IL-27-associated immunoreactivity retained 79.3% of its acute value versus 14.7% for tumour necrosis factor (TNF). p28-directed intervention increased murine 120 h survival from 10% to 45%. **Conclusions**: Human sepsis shows marked IL-27 subunit discordance across producer and receiver compartments while retaining functional sensitivity of the p28-associated axis, defining a molecular-attribution gap between pathway activity and intact heterodimeric IL-27.

## 1. Introduction

Interleukin-27 (IL-27) is an obligate heterodimer of EBI3 and p28 [1] within a cytokine family that reuses subunits [2,3]. EBI3 also pairs with p35 in IL-35 [4,5]. Human and murine IL-27 biology differs: murine p28 can be secreted as IL-30, whereas isolated human p28 is retained and degraded [6]. Thus, detection of a single chain is not equivalent to detection of intact IL-27. Circulating “IL-27” is nevertheless commonly measured by immunoassay, and a meta-analysis of seven studies found that it discriminated septic from non-septic patients with moderate accuracy, with a summary area under the receiver-operating-characteristic curve (AUC) of 0.88 [7]. An anti-IL-27 antibody has also entered randomised phase 2 testing [8], making molecular attribution clinically relevant.

Sepsis is defined by organ dysfunction from a dysregulated host response to infection [9], remains a leading global cause of death [10], and includes reproducible immune endotypes and a transcriptionally defined immunosuppressed monocyte state (MS1) [11,12,13,14,15,16,17]. EBI3 elevation and p28-associated immunoreactivity have been reported separately in sepsis; their cellular coordination is the missing mechanistic link between a heterodimeric cytokine and a single-analyte clinical signal. Resolving that link is central to interpreting IL-27 measurement and to integrating the reported effects of IL-27 blockade in murine sepsis [18,19] with the inflammatory effects of endogenous IL-27 neutralisation in human monocytes [20].

We therefore tested, in cell-resolved human data, whether *EBI3* and *IL-27* are coordinately induced, whether they are co-detected in the same monocytes, and whether receptor-positive T and NK cells show a prespecified IL-27-associated response. Secondary analyses defined the *EBI3*-associated monocyte state and evaluated patient-level discrimination and aggregation effects.

## 2. Materials and Methods

### 2.1. Study Design and Data Sources

The study combines published single-cell and bulk transcriptomes with analyses of two human macrophage datasets, a macrophage-like cell model and murine caecal ligation and puncture (CLP). The principal cohort was GSE216009 (272,993 whole-blood single-cell transcriptomes from 39 individuals: 26 sepsis, seven cardiac surgery, six healthy volunteers, nine paired convalescent samples) with 30 surface markers. The cross-cohort meta-analysis used GSE216009, SCP548 and SCP3740 (166 donor records, two platforms). GSE167363 and GSE175453 provided ratio replication; GSE279451 provided an additional surface-protein replication context; GSE151263 contributed supporting cell-count context. Eight bulk cohorts were used for patient-level analyses and GSE87218 for chromatin accessibility. Two pairs of accessions are arms of single studies rather than independent cohorts and are treated as such: GSE85243 and GSE87218 are the transcriptome and chromatin arms of one experimental series, and GSE279448 and GSE279451 are the bulk and CITE-seq arms of one patient cohort. SCP548 and SCP3740 originate from the same institution and research programme, so donor overlap between them cannot be excluded from the deposited records; the leave-one-cohort-out analysis in Table S5 (Supplementary Materials) shows that the pooled estimate does not depend on treating them as independent.

### 2.2. Single-Cell Processing, Cell Identity and Surface-Protein Normalisation

Cells were filtered and annotated with Seurat v5.2.1 [21], with Harmony v2.0.5 [22] used for annotation and the Section 3.3 embedding. All detection, differential-expression, pseudobulk and meta-analytic models used non-integrated counts within cohort. No clinical label entered the integration.

Surface protein was measured by antibody-derived tags [23]. SoupX v1.6.2 [24] was applied to droplet-based cohorts; the nanowell principal cohort was processed without droplet-based ambient correction because it contains no empty droplets. Principal-cohort subset gates were repeated under centred log-ratio and dsb [25] normalisation. Eleven antibody-derived features in GSE175453 were removed before analysis. Because *EBI3* is also a dendritic-cell gene, the main analysis was repeated after excluding ten dendritic-cell markers. Monocyte subsets were defined by surface CD14/CD16 gates [26,27].

### 2.3. Cross-Cohort Meta-Analysis and Subunit Detection Models

Cross-cohort effects were estimated from donor-level monocyte pseudobulk and pooled by random-effects meta-analysis [28]. The common universe comprised 13,872 genes. DerSimonian–Laird was used for the genome-wide screen; REML with Hartung–Knapp was the confirmatory estimator for *EBI3*. Paule–Mandel, fixed-effect, leave-one-cohort-out and prediction-interval analyses were prespecified sensitivity analyses.

A transcript was detected when at least one unique molecular identifier (UMI) was present; no imputation was used. Between-group detection used logit(P) ~ group + log (UMI) with patient-clustered SEs and t(G − 1) inference. Paired analyses used patient blocking. Ratios of odds ratios (ORs) were estimated from a stacked gene × group model so covariance between the two chains was retained. Pairs in which one chain had no positive cells in the comparator arm were separated from this model and were refitted by Firth penalised likelihood, with profile penalised-likelihood intervals.

### 2.4. Same-Cell Co-Detection and Benchmark Complexes

Same-cell co-detection was defined as joint detection of both transcripts and expressed as an OR against the product of marginal detection odds, with patient-clustered inference. Benchmark pairs were the three C1q combinations and *S100A8*/*S100A9*, selected for obligate or near-obligate complex formation and overlapping detection ranges. *EBI3*/*IL23A* was analysed separately because it contains *EBI3*. Pairs with fewer than ten positive cells in a group were not modelled.

Differential expression used patient-level pseudobulk comparing *EBI3*-positive and *EBI3*-negative septic monocytes. Thirty genes matched to *EBI3* detection were run through the same pipeline as an empirical null. Additional cell-count and sequencing-depth matching tested the effect of arm size and depth. Pathway analysis used MSigDB Hallmark and Reactome collections; regulons used DoRothEA and decoupleR [29,30]. An L1 classifier [31] excluded *EBI3*, *IL-27* and genes correlated with either at |r| > 0.30; folds were assigned by sample.

### 2.5. The EBI3-Associated State and Regulatory Analyses

Differential expression used patient-level pseudobulk, with patient as a blocking factor and sequencing depth as a covariate; patients with fewer than five cells in either arm were excluded. The subset-adjusted model added protein-defined monocyte subset. The unadjusted contrast was treated as a decomposition and the adjusted contrast as the primary subset-aware estimate.

Detection-matched placebo genes, cell-count matching and depth matching were prespecified calibrations. Hallmark comparisons used the fixed 50-set collection; the broader pathway analysis used Hallmark plus Reactome, with one false-discovery-rate (FDR) family.

DoRothEA confidence A–C regulons were scored with decoupleR cross-checking [29,30], excluding both subunits from regulons. The classifier used sample-level cross-validation and the same feature restrictions in every fold.

### 2.6. Receiver-Side, Patient-Level and Dilution Analyses

The receiver-side module comprised 41 genes induced by IL-27 across at least 10 of 12 T/NK subsets in a published single-cell cytokine atlas [32] and was applied unchanged. Module scores used binned control genes. Receptor-positive cells expressed both *IL-27RA* and *IL6ST*; receptor-negative cells expressed neither, with one-chain cells analysed separately. Within-patient contrasts were calibrated against a qualitative IL-15 positive control and 200 detection-matched receptor substitutions. The sensitivity of the module was characterised post hoc by adding an effect of known size to the module score of the dual-receptor-positive cells and refitting the identical patient-clustered model across a grid of effect sizes, taking exclusion of zero by the 95% interval as evidence of detection; the resulting minimum detectable effect is given in Table S17.

Patient-level analyses used one value per individual. Bulk models were composition-adjusted with marker scores [33] and used only for severity, outcome and cross-compartment discrimination. The *EBI3*-to-p28 ratio was calculated as (*EBI3*% + 0.1)/(p28% + 0.1). Four comparators were fixed in advance: healthy volunteers, cardiac surgery, convalescence and pooled non-infected controls. Comparator robustness used the floor and spread of four-arm AUCs, 500 detection-matched null objects and 2000 paired patient bootstraps. Dilution simulations resampled each individual’s cells while reducing monocyte content. The simulation makes no assumption about how either score scales with monocyte content. Rather than applying a dilution model, it resamples each individual’s own cells to a target monocyte fraction at a fixed total cell number and sums the observed counts, so the contribution of non-monocyte cells to each score is measured rather than imposed. The predictions were fixed before the simulation was run and included the differential prediction that MS1 would not be diluted, because its constituent genes are also expressed outside the monocyte compartment; a scaling assumption applied to both scores could not have produced the observed crossing.

### 2.7. Statistical Inference, Missing Data and Software

ORs and ratios of ORs used patient-clustered inference with CR1 correction and t(G − 1) reference distributions [34,35]. AUCs used DeLong intervals and paired tests [36,37]. Multiple testing used Benjamini–Hochberg within stated families. No single overarching correction was applied across the full set of claims. Multiplicity across claims was instead addressed by fixing the primary analyses and their criteria in advance and by calibrating each principal result against a null generated by passing matched objects through the identical pipeline, using detection-matched genes, structure-preserving permutation, matched gene pairs, matched receptor substitutions and placebo genes. This approach calibrates each claim against its own analysis pipeline but does not control a family-wise error rate across the paper. Secreted and cytokine annotations were assigned after ranking. The same pre-frozen complex definition was used for the secreted-proteome analysis. Weighted gene co-expression network analysis (WGCNA, v1.73) was applied to the same pseudobulk profiles.

Missing data were not imputed. Undetected transcripts were treated as zero counts; analyses excluded patients lacking required covariates, outcomes or cell counts. The patient-level *EBI3*-to-p28 ratio used a fixed 0.1 percentage-point offset.

Analyses used R 4.3.3 and Python 3.11; package versions, seeds and full QC thresholds are recorded in the analysis code. Terminology distinguishing molecular attribution from pathway-associated readouts is defined in Table S26 and used consistently throughout.

### 2.8. Experimental Methods

#### 2.8.1. Public Human Macrophage Datasets

GSE85243 and GSE185433 were re-analysed for the response of *EBI3* and *IL-27* to six days of lipopolysaccharide (LPS) preconditioning. Three paired donors per dataset were included. The donor-level interaction Δ*EBI3* − ΔIL-27 was tested by exact sign-flip permutation over all 2^6^ assignments, with both one- and two-sided values reported. The experimental series that follows was designed to test transcript coordination and functional sensitivity of the p28-associated axis across human macrophage and murine systems.

#### 2.8.2. THP-1 Macrophage-like Cell Model and Endotoxin Tolerance

THP-1 cells (ATCC, Manassas, VA, USA; TIB-202) were maintained in RPMI-1640 supplemented with 10% fetal bovine serum, 2 mM L-glutamine, 100 U/mL penicillin and 100 μg/mL streptomycin at 37 °C in a humidified atmosphere containing 5% CO_2_. Cells were differentiated into adherent macrophage-like cells with 25 ng/mL phorbol 12-myristate 13-acetate (PMA; Sigma-Aldrich, St. Louis, MO, USA, P8139) for 72 h, after which the PMA-containing medium was removed, the cells were washed and they were rested for a further 24 h in PMA-free complete medium.

The endotoxin-tolerance protocol followed the established THP-1 macrophage framework of Al-Shaghdali et al. [38]. Three states were generated. Untreated cells received complete medium alone. For acute stimulation, differentiated cells were exposed to 100 ng/mL *Escherichia coli* K12 lipopolysaccharide (LPS; InvivoGen (San Diego, CA, USA), tlrl-eklps) for 18 h. To generate the tolerant-like state, cells were preconditioned with 100 ng/mL K12-LPS for 24 h, the LPS-containing medium was removed, the cells were washed and they were rechallenged with 100 ng/mL K12-LPS for a further 18 h. Cells and conditioned supernatants were harvested at the end of the corresponding period.

#### 2.8.3. Surface HLA-DR Flow Cytometry

Cells were harvested, washed in a PBS-based staining buffer and stained with an APC-conjugated anti-human HLA-DR antibody (clone L243; BioLegend (San Diego, CA, USA), 307610) for 30 min at 4 °C in the dark. Samples were acquired on an LSRFortessa X-20 flow cytometer (BD Biosciences, San Jose, CA, USA) using identical acquisition settings within each experimental batch and analysed in FlowJo 10.8.1 (Tree Star, Ashland, OR, USA).

After exclusion of debris and doublets, the HLA-DR-high population was defined as the discrete HLA-DR-positive population above a fluorescence-minus-one gating boundary. Once established, the gate was fixed and applied identically to every arm within the corresponding batch. The percentage of HLA-DR-high cells was the prespecified surface readout, and three wells were analysed per arm.

#### 2.8.4. Soluble Mediators and Lactate Dehydrogenase Release

Cell-free conditioned supernatants were collected at harvest, clarified by centrifugation and stored frozen until analysis. TNF-α, IL-6 and IL-10 were quantified with commercial human sandwich enzyme-linked immunosorbent assay (ELISA) kits (Proteintech, Rosemont, IL, USA; TNF-α, KE00154; IL-6, KE00139; IL-10, KE00170) according to the manufacturer’s instructions. The IL-6 and IL-10 samples were assayed at a 1:5 dilution and the concentrations were corrected for that factor.

IL-27-associated immunoreactivity was quantified with a human IL-27 ELISA kit (Abcam (Cambridge, UK), ab83695) and interpolated from the plate-specific standard curve. Because this assay reports IL-27 immunoreactivity rather than chain-resolved quantification of EBI3 and p28, the readout is referred to throughout as IL-27-associated immunoreactivity.

Lactate dehydrogenase release was assessed with a Proteintech lactate dehydrogenase cytotoxicity assay kit, with absorbance read at 490 nm on a SpectraMax i3x multi-mode microplate reader (Molecular Devices, San Jose, CA, USA), as a membrane-integrity control. Because no maximum-release condition was included, the measurements were retained as optical-density values and compared between arms within the same plate rather than converted to percentage cytotoxicity. Eight wells were analysed per arm for the soluble readouts.

#### 2.8.5. Bacterial-Association Assay

Association with labelled bacteria was assessed with a pHrodo Green *Escherichia coli* BioParticles conjugate (Invitrogen (Carlsbad, CA, USA), P35366). Lyophilised particles were resuspended at 1 mg/mL in pH-neutral uptake buffer, dispersed before use and added to the differentiated cells at a final concentration of 100 μg/mL. Untreated, acute-LPS and tolerant-like cells were incubated with the particles for 2 h at 37 °C, then washed, fixed with 4% paraformaldehyde and counterstained with 4′,6-diamidino-2-phenylindole.

Because pHrodo Green fluorescence increases on internalisation and acidification within the phagosome, pHrodo-positive nucleated cells were scored. Images were acquired with identical settings on an Axio Imager D2 fluorescence microscope (Carl Zeiss, Oberkochen, Germany) and analysed in Imaris 10.1.0 (Oxford Instruments, Abingdon, UK). Two to five randomly selected fields were acquired from each well and eight wells were analysed per arm; field-level percentages were averaged within each well before analysis, so the well, rather than the field or the individual cell, was the unit of analysis.

#### 2.8.6. In Vitro Perturbation of the p28-Associated Axis

Recombinant human IL-27 (R&D Systems (Minneapolis, MN, USA), 2526-IL) was applied at 100 ng/mL for 16 h before the terminal lipopolysaccharide challenge, with vehicle-treated cells as the corresponding control.

For p28-directed perturbation, tolerant-like cells received 10 μg/mL of a functional-grade monoclonal antibody against IL-27 p28 (clone MM27-7B1; Invitrogen/eBioscience (San Diego, CA, USA), 16-7285-85) during the final challenge period, with an isotype-matched mouse IgG2a κ antibody (clone eBM2a; Invitrogen/eBioscience, 16-4724-85) applied at the same concentration. The recombinant-ligand and antibody experiments were conducted in separate batches and analysed only within their respective batches. Surface HLA-DR and secreted TNF-α were quantified as described above. The supplier lists this clone as reactive with mouse and states that it inhibits the activity of both mouse and human IL-27, which is the basis for its use in this human cell model; neutralisation and inhibition are listed among its reported applications, although the supplier records its species reactivity as mouse and we obtained no independent evidence of functional validation in a human system; the preparation is functional grade at greater than 90% purity by SDS-PAGE. No independent in-house specificity testing was performed beyond the isotype-matched control.

#### 2.8.7. Caecal Ligation and Puncture

Male C57BL/6 mice aged 6–8 weeks and weighing 18–22 g were obtained from Shanghai Yishang Biotechnology Co., Ltd. (Shanghai, China) and were of specific-pathogen-free grade. All animal procedures were performed at the same facility. Animals were maintained under specific-pathogen-free conditions with free access to food and water, housed six per cage under a 12 h light/dark cycle with standard environmental enrichment consisting of nesting material and a shelter, and acclimatised for three days before surgery. Mice were anaesthetised with isoflurane. Under aseptic conditions, a 1 cm midline abdominal incision was made and the caecum was exteriorised. Approximately half of the caecal length was ligated with 4-0 silk without obstructing intestinal continuity, and the ligated caecum was punctured near the ligation site with a 21 G needle. The caecum was returned to the abdominal cavity and the incision was closed. Sham-operated mice underwent the same laparotomy and caecal manipulation without ligation or puncture. Immediately after surgery all mice received sterile saline at 1 mL per 20 g body weight for fluid resuscitation. Buprenorphine 0.1 mg/kg was given subcutaneously before surgery and every 8 h thereafter for 48 h. Predefined humane endpoints were loss of at least 20% of baseline body weight, or loss of at least 15% together with overt clinical abnormality; a body condition score of 2 or less out of 5; persistent inability to eat or drink; marked dehydration; sustained hunching, markedly reduced activity, or inability to ambulate normally or to reach food and water; markedly reduced responsiveness to stimuli or a moribund state; sustained respiratory distress or cyanosis; severe or uncontrolled haemorrhage; wound dehiscence or severe wound infection; and marked uncontrolled pain persisting despite analgesia. Animals reaching any endpoint were euthanised after veterinary consultation by carbon dioxide inhalation followed by cervical dislocation to confirm death. The parameters monitored were body weight, body condition score, food and water intake, activity and behaviour, posture, coat condition, hydration status, respiratory status, signs of pain and the surgical wound.

#### 2.8.8. In Vivo p28-Directed Intervention, Survival and Peritoneal Macrophage Analysis

For p28-directed intervention, mice received a single intraperitoneal injection of 40 μg per mouse of an antigen-affinity-purified goat polyclonal antibody against mouse IL-27 p28/IL-30 (R&D Systems, AF1834) one hour before caecal ligation and puncture; the corresponding control goat immunoglobulin G (IgG) was given at the same time and by the same route. Sham animals provided surgery control. Animals were allocated to groups using a random number table. Because sham and CLP animals underwent different surgical procedures, sham-versus-CLP allocation could not be concealed from the surgeon. For the two CLP intervention groups, treatment identity (anti-p28 versus control IgG) was concealed from the surgeon and from the investigator who assessed the outcomes. No specific strategy was used to control for the order of surgery or for cage location. Survival to 120 h after surgery was the prespecified primary in vivo outcome; the 48 h large-to-small peritoneal macrophage ratio was a secondary outcome. Group sizes were based on previous experience with this model; no a priori power calculation was performed. No inclusion or exclusion criteria were set in advance. Survival was monitored for 120 h with 20 mice per group, with animals observed at 4-hourly intervals; deaths and euthanasia at a predefined humane endpoint were recorded as events at the observation time point at which they were first identified. No animals were excluded from the survival analysis. Expected sepsis-associated morbidity and mortality occurred; no unexpected adverse events were observed. This polyclonal reagent was raised against NS0-derived recombinant mouse IL-27 p28/IL-30 (Phe29–Ser234), antigen-affinity-purified, and is validated by the supplier for neutralisation of recombinant mouse IL-27 bioactivity. Polyclonal preparations carry a higher risk of off-target binding than monoclonal reagents; no independent specificity testing was performed, and the control goat IgG was matched for species and purification.

For peritoneal macrophage composition, a separate cohort was studied 48 h after surgery. At 48 h, mice were euthanised by carbon dioxide inhalation followed by cervical dislocation to confirm death, after which the peritoneal cavity was lavaged with 5 mL of pre-cooled PBS [39]. Recovered cells were washed and incubated with an Fc-blocking reagent (BD Pharmingen (San Jose, CA, USA), 553141) for 15 min at 4 °C, then surface-stained for 30 min at 4 °C in the dark with FITC-conjugated anti-mouse F4/80 (clone BM8; BioLegend, 123108) and PE-Cy7-conjugated anti-mouse I-A/I-E (clone M5/114.15.2; BioLegend, 107630). Samples were acquired on an LSRFortessa X-20 and analysed in FlowJo. After exclusion of debris and doublets, identical F4/80–MHC-II gates were applied across samples, and large and small peritoneal macrophages were resolved as F4/80-high MHC-II-low and F4/80-low MHC-II-high populations respectively [40]. The large-to-small ratio was calculated for each animal. Four mice were allocated to each group (sham, CLP + control IgG and CLP + anti-p28); one control-IgG sample was lost to technical failure during processing, so n = 3 contributed to that analysis. Thus, 12 mice were allocated to the 48 h cohort and 11 contributed data; across the survival and 48 h cohorts, 72 mice were allocated and 71 contributed to the reported analyses. The study protocol was not registered on a public platform; the design, group sizes and outcome measures were specified in advance in the animal ethics application (protocol code YISH-20230227-002).

#### 2.8.9. Statistical Analysis of the Experimental Data

For the in vitro experiments the well was the unit of analysis; microscopy fields were averaged within wells and were not treated as independent observations. Two-group comparisons used Welch’s t test, and three-group comparisons one-way analysis of variance with Tukey’s multiple-comparison procedure. For the in vivo experiments the individual animal was the unit of analysis. Survival was compared by log-rank test, with hazard ratios and 95% confidence intervals from Cox proportional-hazards regression; the proportional-hazards assumption was assessed by scaled Schoenfeld residuals. Peritoneal macrophage ratios used one value per animal.

## 3. Results

### 3.1. EBI3 Ranks 137th and IL-27 9413th Among 13,872 Genes in Septic Monocytes

**Figure 1:**
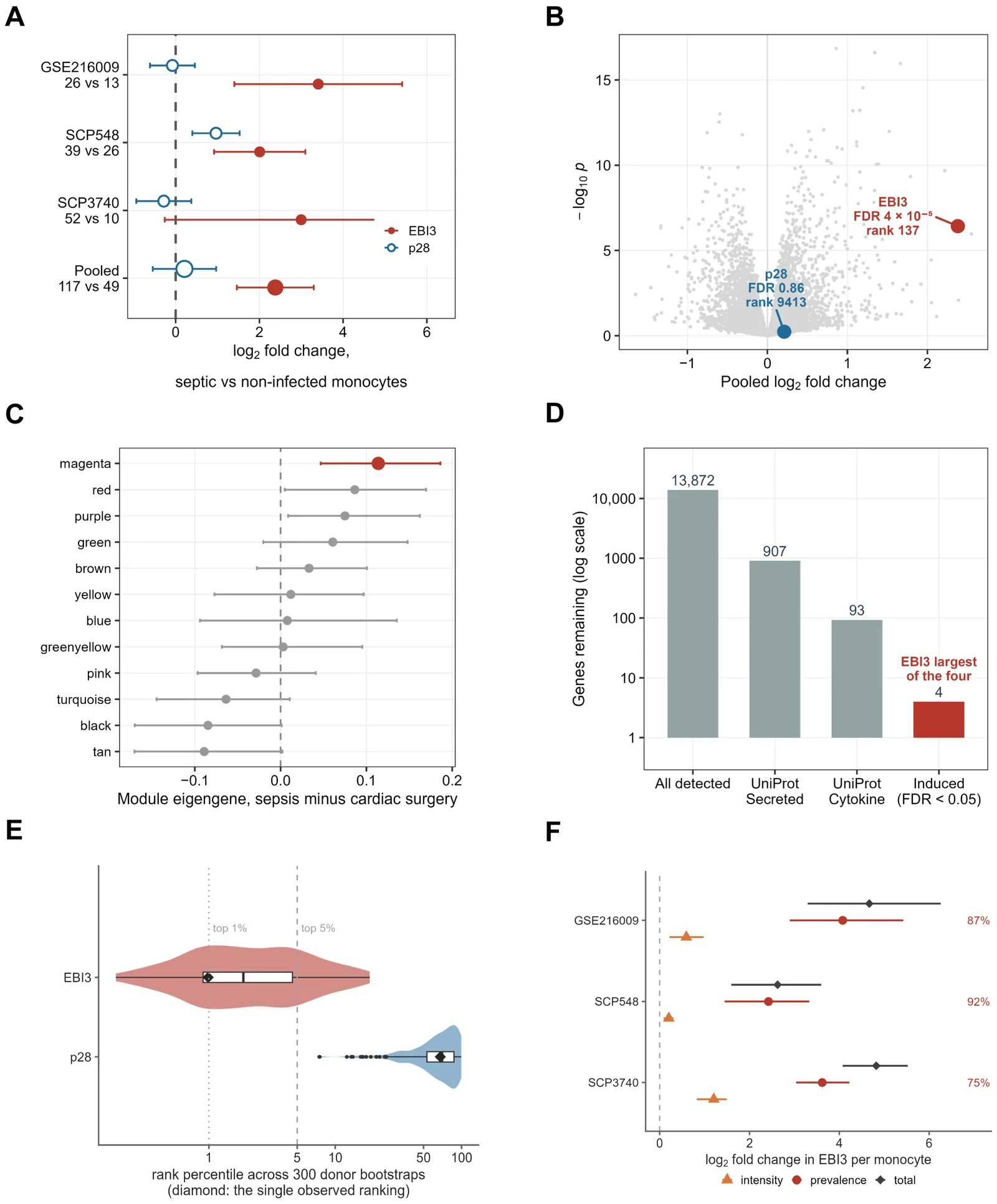
Hypothesis-free nomination of *EBI3*. (**A**) Cross-cohort monocyte meta-analysis (166 donor records). (**B**) Genome-wide ranking of 13,872 shared genes. (**C**) Weighted gene co-expression network analysis (WGCNA) module context. (**D**) Secreted-protein/cytokine annotation. (**E**) Rank stability across 300 donor bootstraps. (**F**) Decomposition of the *EBI3* fold change into a prevalence term (more positive cells) and an intensity term (more transcript per positive cell), in each of the three meta-analysis cohorts; the red value beside each cohort is the prevalence share of the total change. In (**B**), red marks EBI3, blue p28 and grey the remaining genes.

Monocyte pseudobulk profiles from three cohorts (166 donor records, two platforms) were compared between sepsis and non-infected controls (Figure 1A). *EBI3* showed a pooled log2 fold change of +2.38 and ranked 137th/13,872; *IL-27* ranked 9413th with a pooled effect of +0.21 (Figure 1B). Donor bootstrapping placed *EBI3* in the top 5% in 78% of 300 resamples. Co-expression and secreted-proteome ranking independently converged on *EBI3*, while *IL-27* remained far lower in the genome-wide ranking. The confirmatory REML/Hartung–Knapp estimate for *EBI3* was +2.44 (95% CI +0.57 to +4.32; *p* = 0.030).

*EBI3* remained highly ranked when genes were retained by detection rather than bulk-expression filters, avoiding exclusion of low-detection genes. The ranking was stable to alternative meta-analytic estimators and leave-one-cohort-out analyses.

WGCNA on the same donor-level pseudobulk placed *EBI3* in a C1q-rich module (Figure 1C), providing convergent co-expression context for the *EBI3*-associated state.

Detection-matched null calibration placed *EBI3* at the extreme positive tail in the principal cohort and SCP548, whereas *IL-27* showed cohort-restricted elevation. This calibration reinforced *EBI3* as the reproducible cross-cohort signal. Matching on total expression rather than on detection gave the same percentiles, and at *IL-27*’s own expression level reference genes reached detection odds ratios of about 3 at the 97.5th percentile of the matched distribution and up to 18.9 at the maximum. This argues against a global assay floor in that detection regime but does not quantify *IL-27*-specific dropout or guarantee that a given true effect would have been detected (Tables S9 and S10).

Annotation of the ranked 907 secreted proteins placed *EBI3* near the top of the secreted set and among cytokines, while *IL-27* remained low (Figure 1D). Other heteromeric secreted complexes showed coordinated partner-chain ranking, providing a biologically matched benchmark for the IL-27 subunits.

This population-level nomination motivated direct testing of whether *EBI3* and p28 occur in the same cells.

Together, cross-cohort effect-size meta-analysis, donor resampling (Figure 1E), co-expression and secreted-proteome ranking consistently nominated *EBI3* over *IL-27*, establishing a reproducible population-level subunit imbalance for cell-resolved testing.

### 3.2. Discordant IL-27 Subunit Induction Attenuates During Convalescence

**Figure 2:**
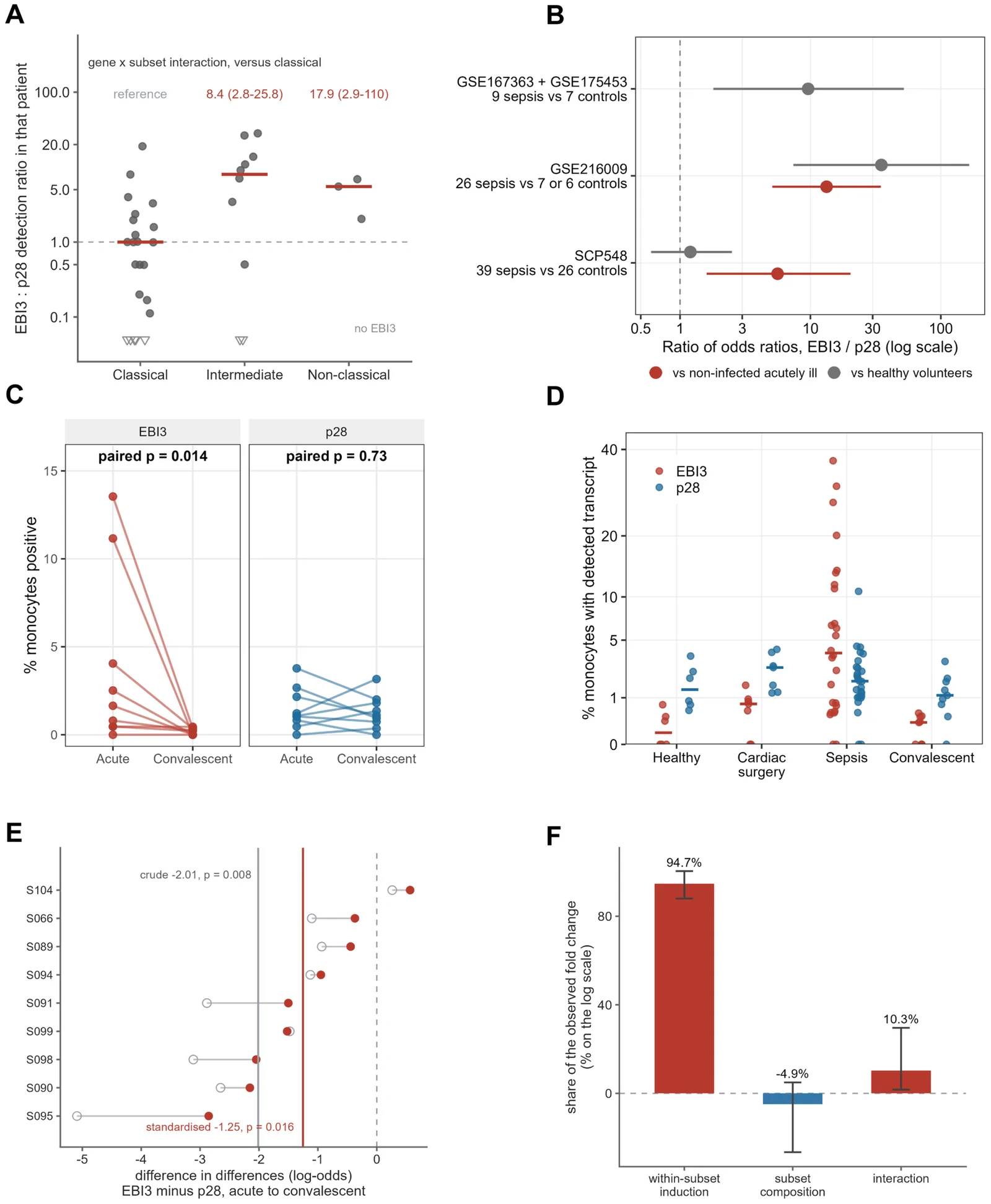
Discordant induction of the IL-27 subunits in septic monocytes. (**A**) *EBI3* and *IL-27* detection across clinical states, one point per sample with the median marked. (**B**) Ratio of disease-associated odds ratios. (**C**) Acute-to-convalescent change in nine patients. (**D**) *EBI3*-to-p28 detection ratio across monocyte subsets, one point per patient. Values above each subset are gene × subset interaction odds ratios against classical monocytes with 95% intervals; red bars are medians; open triangles are patients in whom p28 was detected but *EBI3* was not, a ratio of zero that the logarithmic axis cannot show. (**E**) Patient-level difference-in-differences with and without subset standardisation. (**F**) Decomposition of the same increase in the principal cohort into within-subset induction, subset composition and their interaction, each as a share of the observed fold change on the log scale. In (**E**), grey denotes crude and red subset-standardised values. In (**F**), red bars are positive and blue negative contributions.

Because IL-27 assembly requires both chains, the patient-level comparison was repeated with detection of *EBI3* and p28 in the same monocytes of the principal cohort (Table 1). Against healthy volunteers, *EBI3* rose strongly while p28 changed little. Against post-cardiac-surgery controls, the ORs were 11.0 (4.60–26.33) for *EBI3* and 0.78 (0.50–1.20) for p28, with a ratio of ORs of 13.3 (5.1–34.7; Figure 2A,B).

The same discordance appeared against non-infected intensive-care controls in the replication cohort with enough p28-positive cells (ratio of ORs 5.6, 1.6–20.3). It was driven mainly by prevalence: the prevalence term accounted for 87%, 92% and 75% of the *EBI3* fold change across the three cohorts (Figure 1F). An additional CITE-seq cohort remained directionally concordant.

In nine patients sampled at acute sepsis and convalescence, *EBI3* declined during recovery while p28 remained stable. The gene × time interaction was 0.067 (95% CI 0.010–0.461; *p* = 0.012; Figure 2C), demonstrating attenuation of the imbalance during convalescence.

The *EBI3*-to-p28 ratio was highest in the intermediate monocyte subset (Figure 2D), and subset adjustment with direct standardisation preserved the longitudinal interaction (Figure 2E).

Across protein-defined monocyte subsets, *EBI3* detection was highest in intermediate and non-classical cells, whereas p28 remained low (gene × subset interaction versus classical monocytes: 8.4, 95% CI 2.8–25.8 for intermediate; 17.9, 2.9–110.0 for non-classical). The *EBI3*-to-p28 ratio therefore varies across the monocyte maturation axis while retaining the same direction of discordance.

Together, the longitudinal and subset analyses localise the discordance to the cellular level and show that it persists across the monocyte maturation axis.

### 3.3. EBI3-Positive Monocytes Define an HLA-DR-High State Distinct from MS1

**Figure 3:**
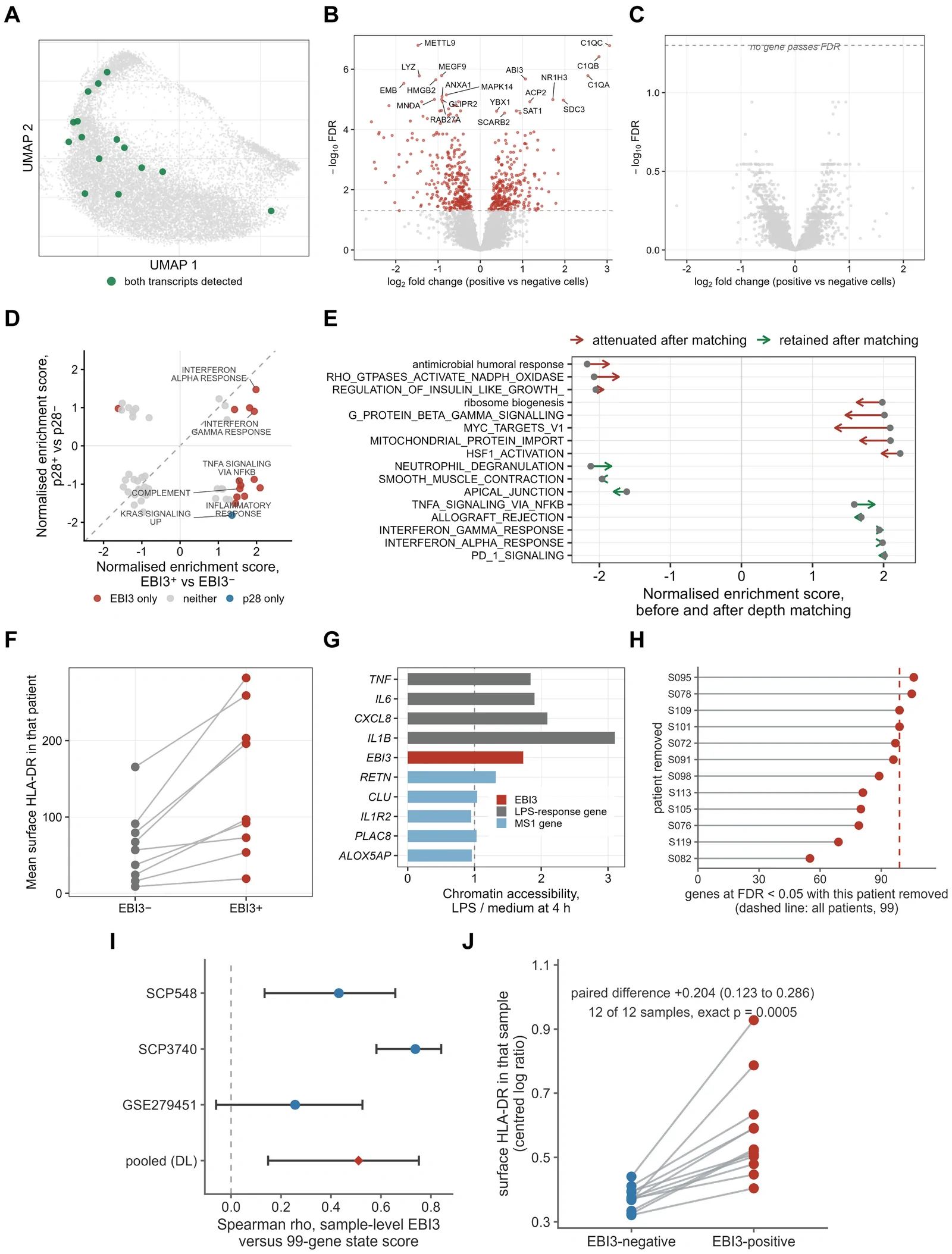
The *EBI3*-associated state is distinct from p28 status and the MS1 monocyte state (MS1). (**A**) Monocytes with both transcripts detected, on the monocyte uniform manifold approximation and projection (UMAP) embedding: 15 of 15,958 monocytes pooled across all clinical states, against 11.6 expected from the marginal detection rates (the septic-only counts, 13 of 7164 against 11.9, are given in Section 3.4). (**B**) *EBI3*-positive versus *EBI3*-negative differential expression. (**C**) Corresponding p28 contrast. (**D**) Hallmark enrichment. (**E**) Depth-matched pathway comparison. (**F**) Surface HLA-DR. (**G**) *EBI3*-locus accessibility after lipopolysaccharide (LPS). (**H**) Leave-one-patient-out signature stability. (**I**) External projection of the 99-gene signature. (**J**) External protein-level replication. In (**A**), green marks monocytes with both transcripts detected and grey all others. In (**I**), blue circles are the individual cohorts and the red diamond the pooled estimate.

Cells carrying both transcripts were rare and scattered across the monocyte embedding (Figure 3A). *EBI3*-positive and p28-positive monocytes mapped to distinct transcriptional programmes: the *EBI3*-positive contrast yielded a substantial differential signature whereas the p28 contrast yielded none (Figure 3B,C). Hallmark enrichment scores were weakly correlated between the two contrasts (Figure 3D), and the *EBI3*-positive state was enriched for inflammatory and interferon-related programmes.

After depth matching, interferon-α/γ response, TNF/NF-κB signalling and inflammatory response remained recurrent features of the *EBI3*-associated state (Figure 3E). Together with subset-aware differential expression, these results define a reproducible transcriptional programme linked to *EBI3* detection and distinct from p28 status.

*EBI3*-positive monocytes were HLA-DR-high, the reciprocal of the defining low-HLA-DR MS1 phenotype (Figure 3F). Within-patient decomposition attributed 94.7% of the *EBI3* detection increase to induction within subsets, against −4.9% for subset composition (Figure 2F). Subset adjustment retained 99 differentially expressed genes and preserved *C1QA*/*C1QC*. Accessibility at the *EBI3* locus rose 1.8-fold after 4 h of lipopolysaccharide in a single-donor chromatin series, alongside canonical response genes and unlike the MS1 markers (Figure 3G); with one donor, this observation is directionally consistent with the transcript data but cannot be generalised to the donor population, and it is not relied upon by any downstream inference. Separately, every leave-one-patient-out refit preserved the direction of all 99 genes (Figure 3H). The frozen 99-gene signature projected positively into all three external cohorts that lack surface protein (pooled Spearman ρ = +0.510, 95% CI +0.148 to +0.752; *p* = 0.008; Figure 3I). An L1 classifier predicted *EBI3* status with cross-validated AUC 0.904 without selecting MS1 markers, and the same AUC was obtained when the MS1-defining genes were excluded from the candidate feature set outright (Table S16). The frozen five-gene MS1 score was lower in *EBI3*-positive monocytes in all twelve evaluable patients. The two states nonetheless co-existed within individuals: both were present in 24 of 26 septic patients and their patient-level frequencies were not inversely related (Spearman +0.145, *p* = 0.48), while within a patient an individual cell tended to carry one state or the other, with MS1-high defined by a median split of the frozen five-gene score. The pooled patient-clustered estimate of that cell-level association did not exclude the null (*p* = 0.114; Table S23). The surface phenotype replicated at the protein level in an independent CITE-seq cohort: in all twelve samples with sufficient cells, *EBI3*-positive monocytes carried more surface HLA-DR (paired difference +0.204, 95% CI 0.123–0.286, exact *p* = 0.0005; Figure 3J).

### 3.4. Rare Same-Cell Co-Detection of EBI3 and IL-27 Matches Chance Expectation

**Figure 4:**
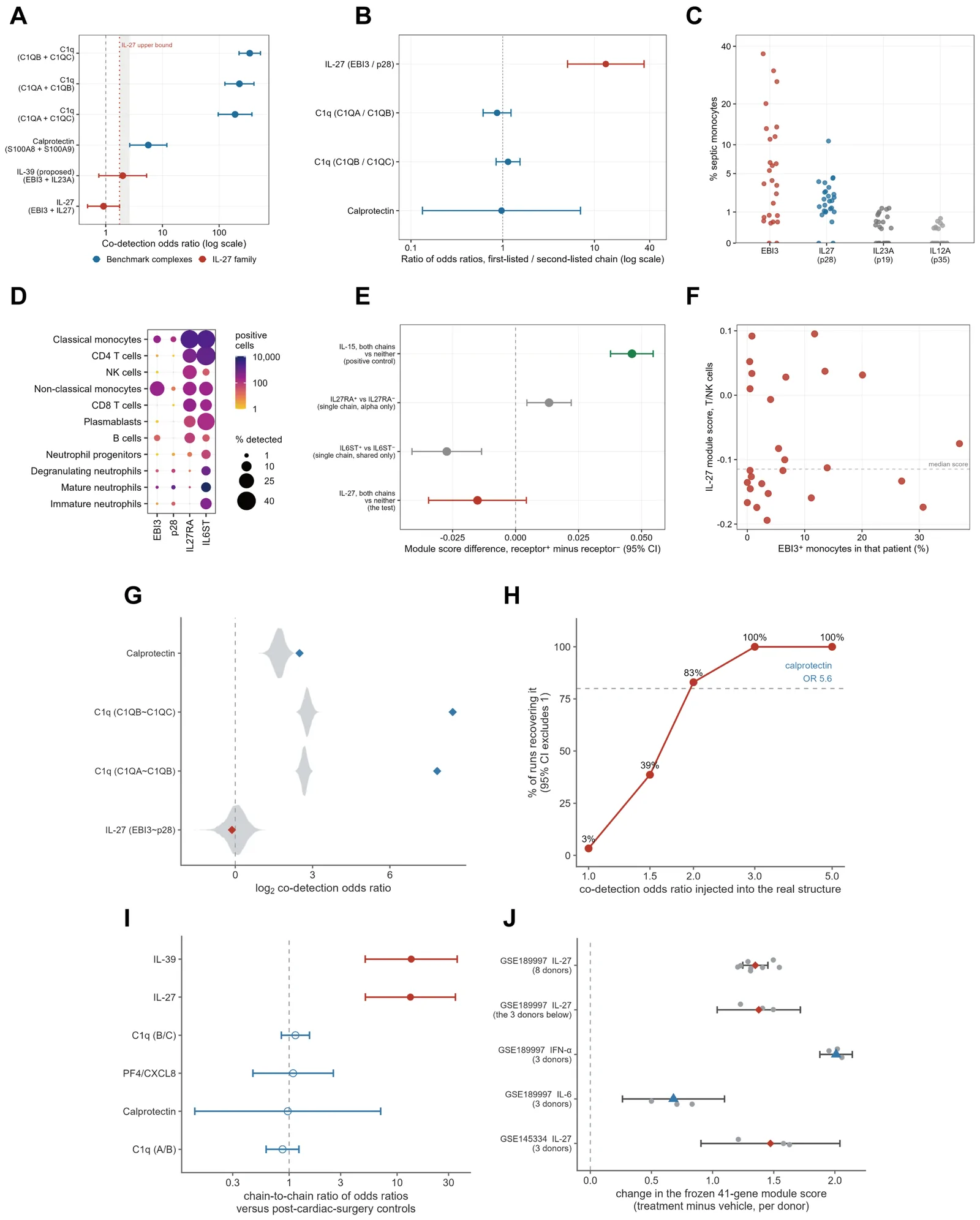
IL-27 subunit discordance persists across same-cell and receiver-side analyses. (**A**) Same-cell co-detection odds for IL-27 and benchmark complexes. (**B**) Chain-to-chain odds-ratio ratios. (**C**) *EBI3* and alternative α-chain detection. (**D**) Ligand and receptor-chain detection across blood cells. (**E**) IL-27-associated module in receptor-positive T and NK cells. (**F**) Patient-level association across monocyte *EBI3* detection. (**G**) Structure-preserving co-detection null. (**H**) Recovery of known associations by simulation. (**I**) Pairwise chain-to-chain contrasts; two pre-frozen pairs are omitted from the panel because a chain has no positive cells in the control arm, which separates the model used here, and are given as Firth penalised-likelihood refits in Table 2. (**J**) Module response to exogenous cytokines. Throughout, red marks EBI3- or IL-27-containing series and blue the corresponding comparator (p28, benchmark complexes, or pairs and arms without EBI3); the green circle in (**E**) is the IL-15 positive control, and grey denotes background or unselected points.

Within septic monocytes, 13 of 7164 cells carried both transcripts, against 11.9 expected from the marginal detection rates, and 97.7% of *EBI3*-positive cells lacked detectable p28. The pooled Fisher OR was 1.11 (*p* = 0.65) and the patient-clustered co-detection OR was 0.91 (95% CI 0.48–1.75). Benchmark complexes in the same cells showed materially higher co-detection (Figure 4A).

A structure-preserving permutation reproduced the same separation. Shuffling *IL-27* detection within patient × subset × depth strata left IL-27 at its structural null: an observed odds ratio of 0.92 against a null median of 1.02, at the 27th percentile of the permutation distribution. The C1q and calprotectin benchmarks gave observed odds ratios of 228.3, 346.7 and 5.63 against null medians of 6.40, 6.91 and 3.24, and exceeded every permutation (Figure 4G). Recovery tests showed that the pipeline detects positive associations when present (Figure 4H).

The benchmark lower confidence bounds were 2.65 or higher, compared with an upper bound of 1.75 for IL-27 (Figure 4B and Supplementary Figure S1). Alternative α-chain analyses reinforced localisation of the discordance to the EBI3–p28 pairing. Against healthy volunteers, where all three EBI3-containing pairs are estimable, the chain-to-chain ratios of ORs were 35.0 (7.4–165.1) for EBI3/p28 (IL-27), 61.6 (10.3–367.8) for EBI3/IL12A and 71.7 (18.9–272.8) for EBI3/IL23A; the corresponding estimate against post-cardiac-surgery controls for EBI3/p28 is the 13.3 reported above (Figure 4I and Table 2).

Alternative α-chain analysis further localised the discordance to the EBI3–p28 pairing (Figure 4C). The cell-resolved data therefore connect the reported diagnostic signal to a previously unrecognised molecular-attribution gap in coordinated EBI3–p28 production.

### 3.5. Receiver-Side Analysis Extends the Discordance Beyond Producer Cells

Receiver-side analysis provided an orthogonal test of IL-27-associated activity. In T and NK cells carrying both *IL-27RA* and *IL6ST*, the prespecified 41-gene module had β = −0.0151 (95% CI −0.0345 to +0.0043; Figure 4D,E). The parallel IL-15 analysis recovered a clear receptor-linked response (+0.0462), confirming the sensitivity of the design to receptor-associated signalling. Detection-matched receptor substitutions placed the observed IL-27 coefficient at the lower edge of the empirical null (95% range −0.0148 to +0.0258).

The dual-receptor definition provided the primary receiver-side estimate; *IL-27RA* alone gave +0.0133 and *IL6ST* alone −0.0273, further distinguishing the joint-receptor analysis from single-chain stratification. At the patient level, the module score did not follow the *EBI3*-positive monocyte fraction across its hundredfold range (Spearman ρ = +0.11, *p* = 0.60; Figure 4F).

Two independent human T-cell cytokine datasets [41,42] confirmed that IL-27 induces the 41-gene module (Figure 4J). Interferon-α and interleukin-6 also induced the programme, supporting its use as an IL-27-associated shared-response module.

### 3.6. The EBI3-to-p28 Ratio Provides Comparator-Stable, Cell-Dependent Discrimination

**Figure 5:**
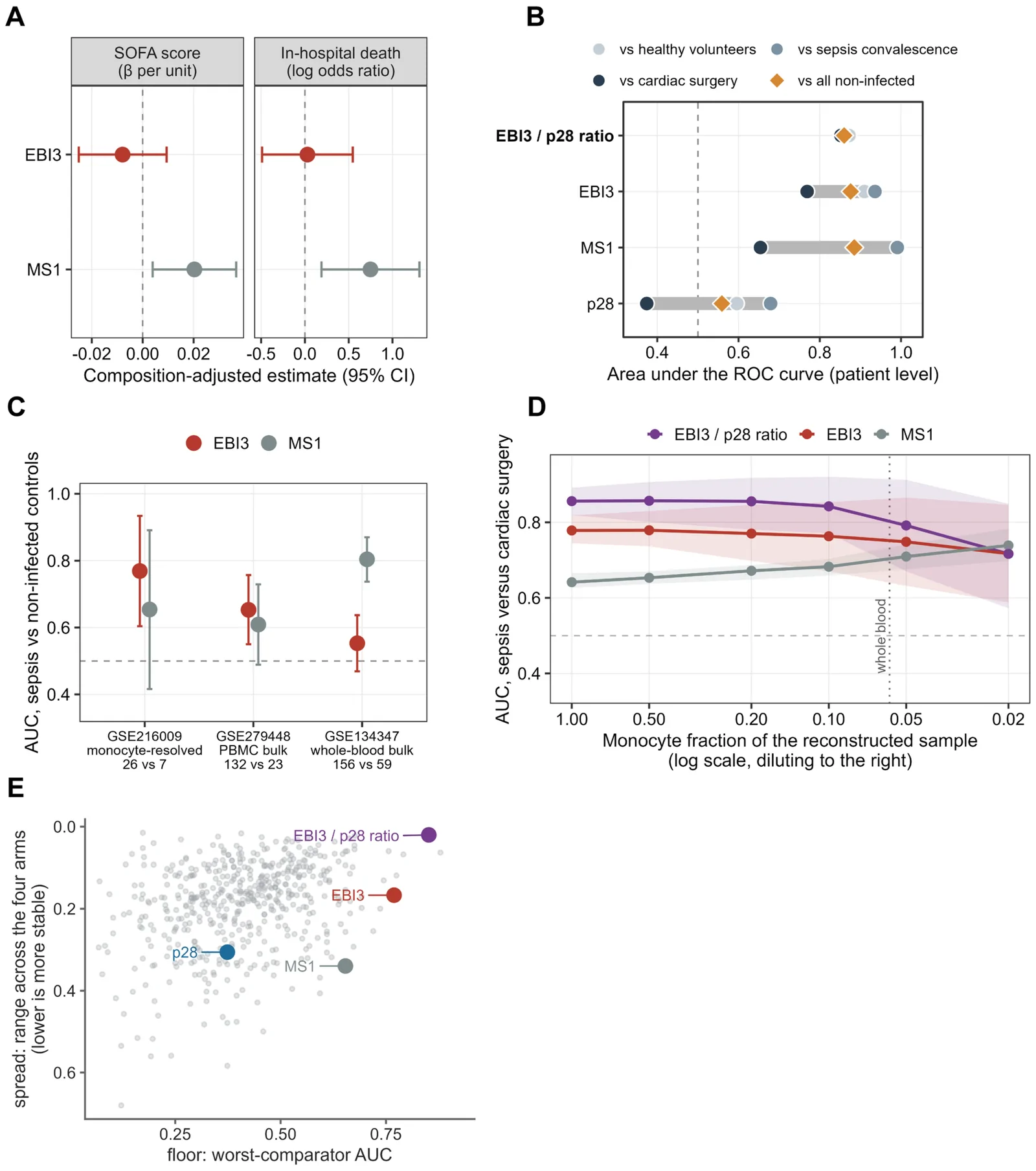
The *EBI3*-to-p28 ratio provides a comparator-stable, cell-dependent signal. (**A**) Associations with Sequential Organ Failure Assessment (SOFA) score and mortality. (**B**) Discrimination across four non-infected comparator groups. (**C**) Discrimination after aggregation to PBMCs and whole blood. (**D**) Dilution with decreasing monocyte content. (**E**) Calibration against 500 detection-matched gene pairs.

Among 348 septic patients, MS1 tracked the Sequential Organ Failure Assessment (SOFA) score (adjusted β = +0.020, *p* = 0.015; n = 345) and mortality (adjusted OR 2.11, 1.21–3.70), while *EBI3* captured an orthogonal dimension of the response (SOFA β = −0.008, *p* = 0.37; mortality OR 1.03, 0.61–1.73; Figure 5A).

Across four comparator arms, the *EBI3*-to-p28 ratio showed the smallest observed spread in patient-level discrimination: AUC 0.852–0.872 (spread 0.020), compared with 0.769–0.936 for *EBI3* and 0.654–0.994 for MS1 (Figure 5B). This is a difference in spread rather than in magnitude: the ratio did not discriminate better than *EBI3* alone in any individual arm (paired DeLong *p* ≥ 0.13), and adding p28 to a model already containing *EBI3* did not improve fit in any arm (likelihood-ratio *p* ≥ 0.13; Table S22). Greater stability than *EBI3* alone was not established by the paired patient bootstrap, whose interval for the difference in spread includes zero (Table S21).

Aggregation changed the scores in opposite directions. *EBI3* discrimination fell from monocytes to peripheral blood mononuclear cells (PBMCs) to whole blood (0.769, 0.653, 0.553), whereas MS1 rose in whole blood (0.654, 0.609, 0.804; Figure 5C). Dilution simulations reproduced the same crossing pattern (Figure 5D). Monocyte content shaped the magnitude of the ratio, while comparator stability was retained. Its combined floor and spread lay beyond the corresponding detection-matched null distribution (Figure 5E).

### 3.7. Functional Validation Connects the p28-Associated Axis to Macrophage State and Survival

The experimental series extended the cell-resolved discovery into human macrophage and in vivo systems, testing whether the subunit imbalance is reproducible and functionally consequential.

**Figure 6:**
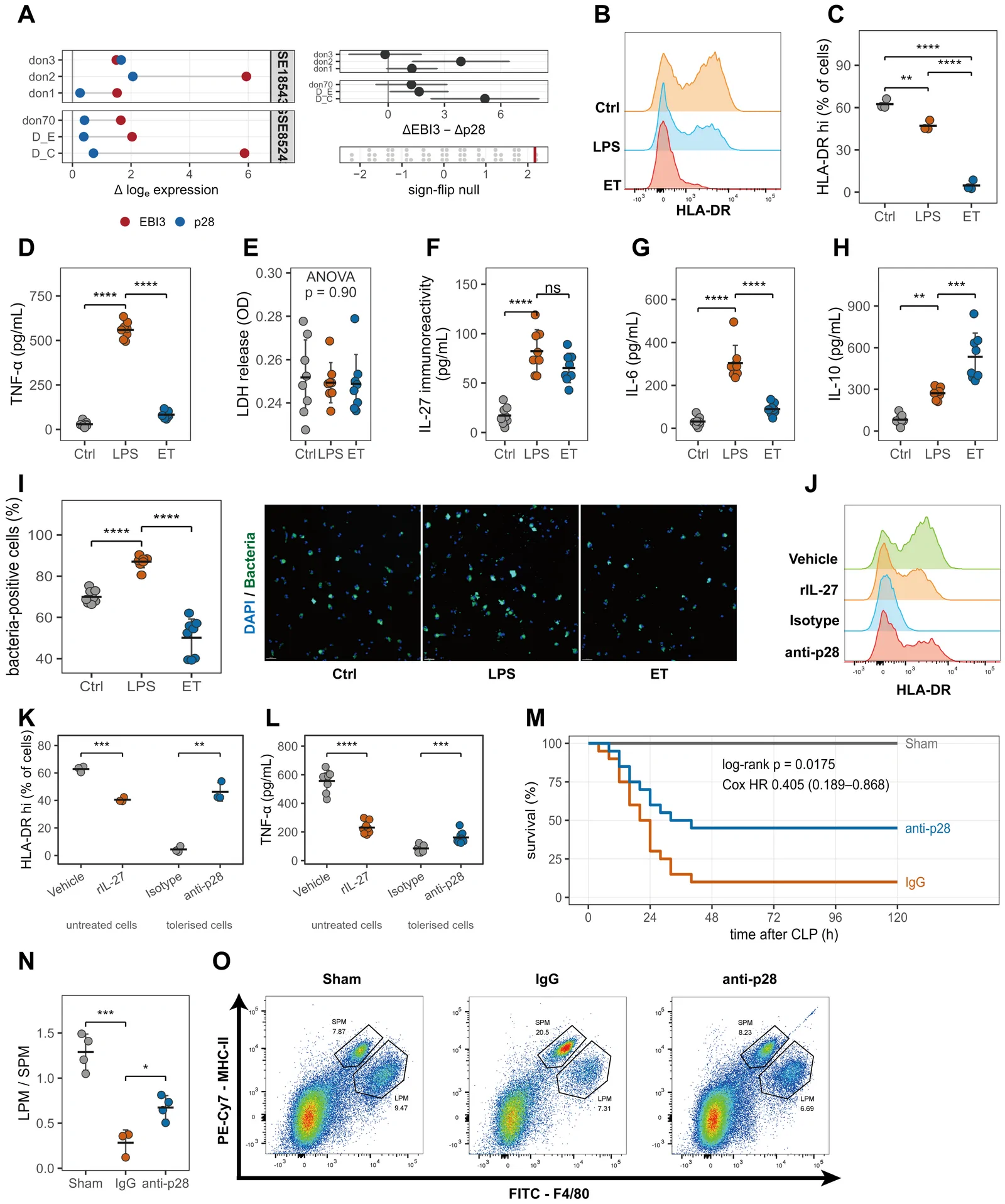
Experimental support for the IL-27-associated axis in human macrophages and mice. (**A**) Paired human macrophage donors after lipopolysaccharide preconditioning: the per-donor change in EBI3 and p28 in two public datasets (GSE185433 and GSE85243; six donors), the within-donor difference ΔEBI3 − Δp28, and the corresponding sign-flip null. (**B**) Surface HLA-DR distributions in the tolerant-like THP-1 model, from which the HLA-DR-high fractions in (**C**) were taken; an identical gate was applied to every arm. (**C**) Surface HLA-DR across untreated, acute lipopolysaccharide and tolerised arms (n = 3 per arm). (**D**) Secreted TNF-α for the same three arms (n = 8 per arm). (**E**) Lactate dehydrogenase release for the same three arms (n = 8 per arm), read as optical density and compared within the plate. (**F**) IL-27-associated immunoreactivity in conditioned medium (n = 8 per arm). (**G**) IL-6 in conditioned medium (n = 8 per arm). (**H**) IL-10 in conditioned medium (n = 8 per arm). (**I**) Cells associated with labelled bacteria (eight wells per arm; the well, not the field or the individual cell, is the unit of analysis), with representative fields (nuclei blue, labelled bacteria green; scale bar 50 μm). (**J**) Surface HLA-DR distributions after recombinant IL-27 in untreated cells and after the p28-directed antibody in tolerised cells, from which the fractions in (**K**) were taken; the same gate was applied throughout. (**K**) Surface HLA-DR for those four arms (n = 3 per arm). (**L**) TNF-α for the same four arms (n = 8 per arm). (**M**) Caecal ligation and puncture (CLP) survival after anti-p28 versus control immunoglobulin G (IgG) (n = 20 per group). (**N**) Peritoneal large-to-small macrophage ratio 48 h after caecal ligation and puncture (sham n = 4, CLP + IgG n = 3, CLP + anti-p28 n = 4). (**O**) Representative peritoneal flow plots for the three arms in (**N**), gated on F4/80 (FITC) against MHC-II (PE-Cy7); the small and large peritoneal macrophage gates are shown with their percentages, one animal per arm. Points are individual wells (in vitro) or animals (in vivo); the horizontal bar is the arm mean and whiskers span ±1 standard deviation (SD). Three-group comparisons are one-way analysis of variance with Tukey’s post-hoc test; the within-pair comparisons in (**K**,**L**) are Welch *t*-tests; (**E**) reports one-way analysis of variance only; (**M**) is a log-rank test with a Cox hazard ratio. * *p* < 0.05; ** *p* < 0.01; *** *p* < 0.001; **** *p* < 0.0001; ns = not significant.

In GSE85243 and GSE185433, six days of LPS preconditioning increased *EBI3* and *IL-27* in all six paired donors, with a positive donor-level Δ*EBI3* − ΔIL-27 interaction in five of six (exact sign-flip *p* = 0.0312 one-sided and 0.0625 two-sided). The interaction estimate was +2.17 on the natural-log expression scale (95% CI −0.21 to +4.55, study-balanced fixed-effect model), reproducing the direction of subunit imbalance in a human macrophage setting (Figure 6A).

In THP-1 macrophage-like cells, LPS preconditioning generated a tolerant-like state. Relative to the acute lipopolysaccharide arm, the tolerised arm had lower surface HLA-DR (47.1% to 4.7%; Figure 6B,C), TNF (559.3 to 82.4 pg/mL; Figure 6D) and IL-6 (304.2 to 89.4 pg/mL; Figure 6G), higher IL-10 (272.2 to 534.4 pg/mL; Figure 6H), and fewer cells associated with labelled bacteria (87.1% to 50.1%; Figure 6I); all five model-validation readouts moved in the expected direction. In the same supernatants, IL-27-associated immunoreactivity remained at 79.3% of its acute value, compared with 14.7% for TNF and 29.4% for IL-6 (Figure 6F).

The IL-27-associated readout therefore remained relatively preserved as the conventional inflammatory programme contracted, linking the transcript-level finding to a distinct secreted signal.

Recombinant IL-27 lowered HLA-DR from 62.9% to 40.5% (*p* = 2.1 × 10^−4^) and TNF from 556.9 to 230.1 pg/mL (*p* = 3.2 × 10^−7^), confirming that the readouts respond to the assembled ligand (Figure 6J–L).

A p28-directed antibody produced the reciprocal response in the tolerant-like arm, increasing HLA-DR from 4.3% to 46.2% (*p* = 0.0047) and TNF from 84.6 to 160.9 pg/mL (*p* = 9.1 × 10^−4^). The reciprocal shifts are shown in Figure 6J–L. Lactate dehydrogenase release remained stable across the experimental arms (Figure 6E and Table S31).

In CLP sepsis, anti-p28 treatment increased 120 h survival from 10% to 45% (log-rank *p* = 0.0175; Cox HR 0.405, 0.189–0.868; n = 20/group; Figure 6M). In a separate cohort, the large:small peritoneal macrophage ratio shifted from 0.28 with control IgG to 0.67 with anti-p28, toward the sham value of 1.29 (ANOVA *p* = 0.0001; treated vs. IgG *p* = 0.033; Figure 6N,O).

Together, the human macrophage and murine experiments connect the transcript-level discovery to functional p28-axis sensitivity, with reciprocal macrophage phenotypes in vitro and improved survival in vivo.

## 4. Discussion

### 4.1. Transcript-Level Discordance as the Central Biological Observation

The central biological advance is the identification of a sepsis-associated uncoupling of IL-27 subunit transcription. *EBI3* and p28 diverge across protein-defined monocyte subsets, the magnitude of divergence exceeds that seen for concordant benchmark complexes, and the imbalance attenuates during convalescence.

The co-detection benchmark is especially informative: in the same cells and abundance range, the lowest benchmark confidence bound was 2.65, whereas the IL-27 upper bound was 1.75. The receiver-side analysis provides a second compartment-level readout, and both human macrophage datasets and the experimental model point in the same direction. Together, the data define a robust transcriptional coordination phenotype.

The phenotype is defined by coordinated divergence across the two chains: *EBI3* induction is reproducible across cohorts, *IL-27* remains largely unchanged across cohorts, and the imbalance diminishes with recovery. Our reanalysis of a published single-cell cytokine-response atlas [43] likewise separates the regulation of the two chains; the original report did not examine EBI3–p28 discordance. The *EBI3*-to-p28 ratio therefore captures a biologically coherent transcript-level coordination phenotype across clinical states and monocyte subsets.

Unpaired EBI3 is biologically plausible: activated human neutrophils release EBI3 without its heterodimeric partners [44], it has chaperone-like activity [45], and it binds interferon-γ and IL-10 [46]. These observations make unpaired EBI3 a plausible contributor to the sepsis-associated signal and motivate direct molecular resolution.

### 4.2. The EBI3-Associated Monocyte Phenotype Is Distinct from MS1

The *EBI3*-associated state is a stimulus-responsive programme embedded within conventional monocyte subsets. Within-subset induction accounted for 94.7% of the increase in *EBI3* detection; subset adjustment retained 99 genes and *C1QA*/*C1QC*. *EBI3*-positive cells were HLA-DR-high, distinct from MS1, and the transcriptome predicted *EBI3* status with AUC 0.904 without selecting MS1 markers.

The phenotype therefore represents an inducible functional state across conventional monocyte differentiation, separable from MS1 and independently reproduced at the surface-protein level.

### 4.3. Orthogonal Evidence from the Producer and Receiver Compartments

Three cell-resolved layers define the attribution problem in sequence. At the producer level, strong *EBI3* induction was unmatched by *IL-27*; at the same-cell level, co-detection remained at chance and below the benchmark range of concordantly transcribed complexes; and at the receiver level, the dual-receptor module estimate remained near zero while IL-15 responsiveness was preserved. Producer, same-cell and receiver analyses therefore converge on the same molecular-attribution gap.

### 4.4. Functional Activity and Molecular Attribution Are Distinct Dimensions of the IL-27 Axis

The patient data show discordant subunit transcription and low same-cell co-detection, whereas p28-directed intervention improves survival in murine sepsis. Together, these findings separate two biologically relevant dimensions of the IL-27 axis: functional sensitivity to p28-directed perturbation and molecular attribution of the circulating signal.

The murine survival result establishes the biological consequence of p28-directed perturbation, while the human cell-resolved analyses define the molecular context in which that activity should be interpreted. This separation of functional sensitivity from molecular attribution is a central conceptual advance of the study. That inference is qualified by a species difference in subunit biology introduced above: murine p28 can be secreted autonomously as IL-30, whereas isolated human p28 is retained and degraded. A p28-directed antibody therefore engages a free-ligand pool in the mouse that the human compartment is not expected to contain to the same degree, so the murine experiment supports functional sensitivity of the p28-associated axis without implying that the same molecular species carries the human signal. The murine experiment is not offered as a new demonstration that p28-directed blockade improves survival in sepsis; that effect is established [18,19]. Its role here is to serve as an existence proof placed alongside the human transcript analyses within one study: it shows that functional sensitivity to p28-directed intervention can be present in the same disease in which coordinated heterodimer production is not evident, and that coexistence is what defines the attribution gap. The corresponding constraints on group size are given in Section 4.6.

The same framework extends to diagnostics: clinical performance and molecular attribution are complementary properties, and cross-subunit assay design provides the direct bridge between them.

### 4.5. Consequences for Biomarker Measurement and Translation

Clinical performance and molecular identity are related but distinct properties of a biomarker. Performance depends on comparator, compartment and assay epitope. The *EBI3*-to-p28 ratio was more stable across non-infected comparators than MS1, while aggregation diluted the monocyte-resolved *EBI3* signal and shifted it in the opposite direction from MS1. Translation therefore depends on cell context as well as assay design. In-silico dilution showed that the ratio, like *EBI3* itself, loses discrimination as monocyte content falls, so it is a within-monocyte quantity; measuring it clinically would require a monocyte-resolved rather than a whole-blood measurement.

A meta-analysis of circulating IL-27 protein reports an AUC of 0.88 for sepsis diagnosis [7], while the discovery programme was anchored to EBI3 [47] and the assay platform measures p28 [48]. Cross-subunit capture/detection, with antibodies recognising different chains, directly assigns signal to assembled IL-27 and provides the clearest route to link diagnostic performance to the heterodimer. That meta-analysis does not itself report the assay platform for any of its seven contributing studies. Three of the seven come from the programme cited here, which used a magnetic-bead multiplex assay and stated that the assay detects the p28 subunit [48]; for the remaining four, the assays are not recoverable from the meta-analysis, and we therefore do not claim that the chain-specificity issue generalises to them.

The intervention literature is also chain-dependent: murine blockade improves outcome [18,19], whereas neutralising endogenous IL-27 in human monocytes increases inflammatory output [20]. Taken together, these studies reinforce the need to interpret pathway activity and molecular attribution as distinct dimensions across species.

### 4.6. Limitations

This study has several limitations. The human analyses resolve subunit coordination at the level of single-cell transcripts and do not establish the molecular composition of the circulating protein signal. Cross-subunit measurement of assembled IL-27 alongside total EBI3 and p28 remains the direct test of the attribution framework, and the immunoassay used here has undefined chain specificity, which is why its output is reported throughout as IL-27-associated immunoreactivity rather than as IL-27. Same-cell, same-timepoint co-detection is an informative upstream indicator but is neither necessary nor sufficient for heterodimer assembly: translated protein can persist beyond its transcript, and secretion need not be synchronous with transcription, so its absence does not exclude asynchronous or trans-cellular pairing. The argument therefore does not rest on co-detection by itself but adds a receiver-side readout, which lies downstream of assembly, secretion and receptor engagement and is not subject to that objection. In the same vein, no chain-specific immunoassay, immunoblot or mass-spectrometric confirmation of the intact heterodimer was performed, so the retained immunoreactivity in tolerant-like cells cannot distinguish assembled IL-27 from free EBI3 or other cross-reactive material. This uncertainty is intrinsic to the molecular-attribution question addressed here and limits what the tolerance experiment can establish; chain-specific quantification is required for direct molecular resolution.

The experimental series is small. Each macrophage dataset contributes three paired donors; the donor-level interaction was positive in five of six and met the prespecified one-sided sign-flip threshold, but its effect-size interval includes zero and the two-sided value is 0.0625. The tolerant-like model uses an established cell line rather than primary cells. In the bacterial-association assay the tolerised arm yielded fewer scored cells per well than the other arms, so a contribution of cell density to that difference cannot be excluded, and chromatin accessibility was assessed in a single donor.

The antibody was given before induction, so the murine experiment tests prophylaxis rather than treatment of established sepsis, and the single 48 h harvest cannot separate prevention of the peritoneal macrophage shift from its reversal. The intervention establishes that the axis is functionally consequential but cannot assign the neutralised species: murine p28 is secreted autonomously as IL-30, whereas isolated human p28 is retained and degraded, and a p28-directed antibody binds p28 whether free or paired; therefore, the in vivo result constrains pathway activity rather than heterodimer identity. This is the same attribution limit that the human data describe, encountered here in the intervention itself. Only male mice were used, so the well-documented sex differences in the immune response to sepsis were not addressed and neither the survival nor the peritoneal result should be assumed to extend to females. The peritoneal endpoint rests on three to four animals per group, which is small for a three-group analysis of variance and leaves that comparison with wide uncertainty; group sizes throughout the in vivo work were set from previous experience with the model rather than from a power calculation.

The receiver-side result is a calibrated negative rather than an equivalence claim, since no smallest effect of interest was defined in advance for that module; a post-hoc characterisation places its minimum detectable effect at 0.0346 module-score units, three-quarters of the in vivo IL-15 response measured in the same cells, so a smaller response cannot be excluded. The *EBI3*-to-p28 ratio is accordingly presented as a comparator-stable readout rather than as a superior classifier: at this sample size, the p28 denominator contributes no detectable independent discrimination, which is the expected consequence of the near-null p28 induction that the study reports. The study reports a large number of estimators, normalisations, benchmark comparisons and permutation calibrations. Although the primary analyses and their criteria were fixed in advance and no overarching correction was applied across claims (Section 2.7), the volume of parallel analyses is itself a source of researcher degrees of freedom, and we note it as such; the direction of the principal findings was preserved across all of them. Finally, the contributing cohorts differ in platform, control definition and sampling time; the discordance phenotype was nonetheless consistent across them and across the prespecified sensitivity analyses.

## 5. Conclusions

In human sepsis, *EBI3* and p28 are discordantly induced and rarely co-detected in the same monocyte. Against post-cardiac-surgery controls, *EBI3* detection had OR 11.0 versus 0.78 for p28, ratio of ORs 13.3. The IL-27 co-detection upper bound was 1.75, below the benchmark lower bound of 2.65, and the receiver-side module estimate was β = −0.0151 (95% CI −0.0345 to +0.0043).

The imbalance was reproduced in human macrophages, IL-27-associated immunoreactivity remained relatively preserved in a tolerant-like state, and p28-directed intervention improved murine survival, establishing functional relevance of the p28-associated axis.

Together, these findings identify subunit discordance as a previously unrecognised feature of the IL-27 axis in human sepsis and expose a molecular-attribution gap between pathway-associated activity and attribution to intact heterodimeric IL-27. Cross-subunit measurement of assembled IL-27 alongside total EBI3 and p28 provides the direct molecular test of this framework.

## Figures and Tables

**Table 1:** Baseline characteristics of the principal cohort (GSE216009). The convalescent column comprises second time points from nine of the twenty-six septic patients; the cohort therefore contains 39 individuals in 48 samples. IQR, interquartile range.

Characteristic	Sepsis	Cardiac Surgery	Convalescent	Healthy
Samples, n	26	7	9	6
Age, median (IQR), y	65 (52–76)	64 (52–70)	68 (56–76)	69 (61–74)
Female sex, n (%)	10 (38%)	2 (29%)	3 (33%)	3 (50%)
Monocytes per sample, median (IQR)	244 (137–416)	308 (252–505)	399 (280–452)	428 (345–492)
Infection source: respiratory/biliary/urinary/abdominal/other	9/5/5/3/4	—	—	—

**Table 2:** Chain-to-chain ratios of odds ratios for the eight pre-frozen complexes under both non-infected comparators. Values above 1 indicate stronger induction of the first-listed chain. Two pairs have no positive cells for one chain in the post-cardiac-surgery arm, which separates the patient-clustered model; they are refitted by Firth penalised likelihood and marked †. Both penalised intervals are wide and neither excludes unity, so these two entries are individually uninformative. On point estimates the separation is nonetheless complete under both comparators: every *EBI3*-containing pair exceeds every pair without *EBI3* (1.85–13.5 versus 0.29–1.14 against post-cardiac-surgery controls). Full model output is in Table S12.

Complex (First-Listed/Second-Listed Chain)	vs. Post-Cardiac Surgery	vs. Healthy Volunteers
IL-27 (EBI3/p28)	13.3 (5.1–34.7)	35.0 (7.4–165.1)
IL-35 (EBI3/IL12A)	1.85 (0.014–15.78) †	61.6 (10.3–367.8)
IL-39 (EBI3/IL23A)	13.5 (5.1–36.1)	71.7 (18.9–272.8)
C1q (C1QA/C1QB)	0.87 (0.61–1.23)	0.65 (0.46–0.91)
C1q (C1QB/C1QC)	1.14 (0.85–1.55)	0.72 (0.47–1.12)
Calprotectin (S100A8/S100A9)	0.97 (0.13–7.05)	0.92 (0.18–4.76)
PF4/CXCL8	1.09 (0.46–2.57)	1.17 (0.58–2.36)
CCL2/CCL8	0.29 (0.002–4.81) †	0.27 (0.04–1.90)

## Data Availability

All transcriptomic data analysed are publicly available under the accession numbers given in Section 2.1 and Supplementary Sections S1 and S7; raw public data are not redistributed here and remain with the repositories cited there. The analysis code, the intermediate result tables underlying every figure and table, the final figure files, the raw laboratory workbooks and the dependency lockfiles are available on request from the corresponding author. No standalone software package was developed; the analysis scripts are the code of record and are retained with their versioned dependencies.

## Supplementary Materials

The following supporting information can be downloaded at: https://www.mdpi.com/article/10.3390/biomedicines14092035/s1, Section S1: Cohort replication and longitudinal support; Section S2: Single-cell object reconstruction and gating validation; Section S3: Cross-cohort nomination and robustness; Section S4: Same-cell co-detection and benchmark calibration; Section S5: The EBI3-associated transcriptional state; Section S6: Receiver-side calibration and positive-control validation; Section S7: Patient-level and cross-compartment validation; Section S8: Reproducibility, module definition and dataset roles; Section S9: Experimental validation: per-sample and per-mouse values.
