# Supplementary material for: Discordant Interleukin-27 (IL-27) Subunit Transcription in Human Sepsis Exposes a Molecular-Attribution Gap
Source: Biomedicines. 2026 Sep 10;14(9):2035. doi: 10.3390/biomedicines14092035 (PMC13604127; doi:10.3390/biomedicines14092035)
Supplement: Supplementary file 1 [file biomedicines-14-02035-s001.zip › biomedicines-4550380-supplementary.pdf]

Supplementary Material

Discordant Interleukin-27 (IL-27) Subunit Transcription in Human Sepsis Exposes a Molecular-Attribution Gap

This supplement provides the validation analyses and source-level results supporting the main text: cohort and longitudinal replication (S1); single-cell object reconstruction and gating validation (S2); cross-cohort nomination and robustness (S3); same-cell co-detection and benchmark calibration (S4); the EBI3-associated state (S5); receiver-side calibration and positive-control validation (S6); patient-level and cross-compartment validation (S7); reproducibility and dataset roles (S8); and experimental validation values (S9).

Contents

Section	Content
S1	Cohort replication and longitudinal support
S2	Single-cell object reconstruction and gating validation
S3	Cross-cohort nomination and robustness
S4	Same-cell co-detection and benchmark calibration
S5	The EBI3-associated transcriptional state
S6	Receiver-side calibration and positive-control validation
S7	Patient-level and cross-compartment validation
S8	Reproducibility, module definition and dataset roles
S9	Experimental validation: per-sample and per-mouse values

Tables S1 and 2 cohort and longitudinal replication · S3 object reconstruction and gating validation · S4 and S5 meta-analysis and leave-one-cohort-out · S6 rank stability · S7 and S8 empirical null and prevalence/intensity decomposition · S9 and S10 expression-matched null and detection-sensitivity calibration · S11 and S12 co-detection benchmarks · S13 subset-aware state · S14 leave-one-patient-out stability · S15 external projection · S16 classifier with MS1 genes hard-excluded · S17 minimum-detectable-effect calibration of the receiver-side module · S18–S20 patient-level and cross-compartment analyses · S21 comparator-invariance calibration · S22 formal comparison of the ratio with each subunit · S23 EBI3-positive and MS1-high co-occurrence · S24 module, S25 dataset roles and S26 terminology · S27–S33 experimental validation values including viability and tolerance-model controls.

Figure S1. Detection-regime calibration and dual-reference concordance.

S1–S2 provide replication and object-validation analyses; S3 supports the cross-cohort nomination; S4 calibrates the same-cell result; S5 validates the EBI3-associated state; S6 validates the receiver-side analysis; S7 provides patient-level and cross-compartment calibration; and S8–S9 provide reproducibility and source-level experimental values.

S1. Cohort replication and longitudinal support

GSE167363 included 7 donors (2 healthy, 5 Gram-negative sepsis). Septic donors were sampled at 0 and 6 h after recognition. Because septic libraries were deeper, all cells were thinned to 3,000 UMI before comparison.

Table S1. GSE167363 detection by group after binomial thinning to 3,000 UMI per cell.

Group	Samples	Monocytes gated	Retained after thinning	EBI3	p28
Healthy	2	3,586	2,664	0.00–0.14%	0.08–0.62%
Sepsis, 0 h	5	5,142	4,162	3.45–27.16%	0.23–6.17%
Sepsis, 6 h	5	7,668	6,395	4.00–27.68%	0.21–6.92%

Detection was calculated on the retained cells; libraries below 3,000 UMI were excluded because they could not be thinned to the common depth.

After depth matching, the EBI3:p28 detection ratio was 39.7, closely matching 35.0 in the principal cohort. Both sampling points were acute, providing an independent ratio-replication context.

GSE279451 showed directionally concordant EBI3 elevation (0.50% in sepsis versus 0.35% in health), extending the comparison to an additional CITE-seq cohort.

Table S2. Longitudinal gene-by-time interaction under nested specifications and direct standardisation in the nine paired patients.

Specification	Cells	Interaction odds ratio	95% interval	*p*
Crude, all monocytes	6,362	0.067	0.010–0.461	0.012
Crude, classifiable cells only	4,569	0.057	0.008–0.409	0.010
Subset-adjusted, classifiable cells only	4,569	0.055	0.007–0.431	0.012

Direct standardisation to the acute-arm subset composition preserved the longitudinal interaction at every pseudocount, demonstrating robustness to monocyte-subset composition.

Pseudocount	Crude patient-level mean	Standardised patient-level mean	Same direction	Exact sign-flip *p* (two-sided)
0.1	−2.93	−2.20	8 of 9	0.008
0.5	−2.01	−1.25	8 of 9	0.016
1.0	−1.62	−0.90	8 of 9	0.020

All quantities were analysed on the log-odds scale; the exact permutation enumerated all 512 sign assignments.

S2. Single-cell object reconstruction and gating validation

Eleven antibody-derived features were removed from the GSE175453 RNA matrix before object reconstruction. The rebuilt object sharpened cell identity while preserving a large sepsis-associated EBI3 effect (OR 43.0) and was used for all main analyses.

Table S3. GSE175453 estimates before and after removal of antibody-derived features and object rebuilding.

Estimate	Original object	Rebuilt object after removal of antibody-derived features
EBI3 odds ratio, sepsis vs healthy (monocytes)	133.5	43.0
Patient-level Wilcoxon p (single cohort)	0.0159	0.0876
Monocyte contribution to $EBI3^{+}$ cells	76.9%	56.1%
Adjusted Rand index against original annotation	—	0.796

Object reconstruction reduced dendritic-cell carryover into the monocyte gate; all main-text results use the rebuilt object.

Monocytes were defined by published annotations where available; otherwise by myeloid-marker fraction >2% with CD14 detected. The stringent CD14-based gate excluded platelet-monocyte complexes and doublets and was retained across analyses.

S3. Cross-cohort nomination and robustness

With three cohorts, pooled EBI3 induction remained positive across estimators. The REML/Hartung-Knapp estimate was +2.444 (95% CI +0.569 to +4.319; p = 0.030). Leave-one-cohort-out estimates remained +2.11 to +3.29. The prediction interval was +1.20 to +3.68 for EBI3 and −1.19 to +1.61 for IL27.

Table S4. Pooled monocyte log2 fold change under four estimators, including REML with Hartung-Knapp.

Estimator	*EBI3*	95% CI	*p*	*IL27*	95% CI	*p*
DerSimonian-Laird	+2.380	+1.462 to +3.299	3.8×10^{-7}	+0.210	−0.547 to +0.968	0.59
REML	+2.444	+1.403 to +3.485	4.2×10^{-6}	+0.210	−0.546 to +0.967	0.59
Paule-Mandel	+2.380	+1.462 to +3.299	3.8×10^{-7}	+0.210	−0.546 to +0.966	0.59
Fixed effect	+2.380	+1.462 to +3.299	3.8×10^{-7}	+0.235	−0.099 to +0.570	0.17
**REML + Hartung-Knapp**	**+2.444**	**+0.569 to +4.319**	**0.030**	+0.210	−1.450 to +1.871	0.64

EBI3 retained positive induction across estimators and every leave-one-cohort-out analysis (+2.11 to +3.29), while IL27 remained comparatively stable across cohorts (Table S5).

Table S5. Leave-one-cohort-out sensitivity of the pooled monocyte log2 fold change (DerSimonian-Laird).

Excluded cohort	*EBI3*	95% CI	*p*	*IL27*	95% CI	*p*
GSE216009	+2.108	+1.074 to +3.141	6.4×10^{-5}	+0.353	−0.870 to +1.575	0.57
SCP548	+3.294	+1.588 to +5.001	1.6×10^{-4}	−0.159	−0.574 to +0.256	0.45
SCP3740	+2.444	+1.174 to +3.713	1.6×10^{-4}	+0.440	−0.580 to +1.460	0.40

Table S6. Stability of the genome-wide ranking under 300 stratified donor bootstraps.

	EBI3	*IL27*
Rank in the single observed analysis	137 / 13,872	9,413 / 13,872
Median bootstrap rank	256	9,608
2.5th–97.5th percentile of rank	36–2,006	2,320–13,433
Median rank percentile	top 1.88%	top 70.2%
Reached the top 1%	29.7% of replicates	0.0%

	EBI3	*IL27*
Reached the top 5%	78.3%	0.0%
Reached the top 10%	93.0%	0.7%
Median pooled log_2 fold change	+2.55 (+1.87 to +3.25)	+0.21 (−0.12 to +0.61)

EBI3 ranked above IL27 in all 300 donor bootstraps; it entered the top 5% in 78.3% of replicates and the top 1% in 29.7%.

Table S7. Detection-matched empirical null for the EBI3 and IL27 detection effects.

Cohort	Gene	Non-septic detection	Septic detection	Odds ratio	Matched genes	Percentile	One-sided empirical *p*
GSE216009	*EBI3*	0.198%	7.859%	39.1	—	99.9th	0.0008
GSE216009	*IL27*	—	—	—	—	69.9th	0.30
GSE216009	*IL12A*	—	—	—	—	38.2nd	0.62
GSE216009	*IL23A*	—	—	—	—	14.7th	0.85
SCP548	*EBI3*	0.331%	2.367%	7.21	1,271	99.8th	0.0016
SCP548	*IL27*	0.501%	1.394%	2.79	1,345	98.1st	0.019

IL27 was also elevated against its detection-matched null in SCP548, motivating the EBI3:p28 ratio as the cross-cohort contrast.

Table S8. Decomposition of the EBI3 and IL27 fold changes into prevalence and positive-cell intensity.

Cohort	Gene	Control cells (positive)	Total log_2FC	Prevalence term	Intensity term	Prevalence share
GSE216009	*EBI3*	5,149 (24)	+4.67	+4.08 (2.90–5.43)	+0.59 (0.22–0.98)	87%
SCP548	*EBI3*	5,272 (29)	+2.63	+2.42 (1.44–3.33)	+0.20 (0.09–0.31)	92%
SCP3740	*EBI3*	11,042 (105)	+4.82	+3.62 (3.04–4.23)	+1.20 (0.83–1.49)	75%
GSE216009	*IL27*	5,149 (105)	+0.03	+0.05 (−0.52–0.69)	−0.02 (−0.23–0.18)	—
SCP548	*IL27*	5,272 (29)	+1.39	+1.37 (0.56–2.26)	+0.02 (0.00–0.03)	—
SCP3740	*IL27*	11,042 (974)	−0.61	−0.77 (−1.30–0.25)	+0.16 (−0.03–0.33)	—

Prevalence accounted for 87%, 92% and 75% of the EBI3 fold change across the three cohorts, respectively.

Table S9. Baseline detectability of each chain in healthy monocytes, and the expression-matched empirical null for the sepsis-versus-health detection effect.

Gene	Detection in healthy monocytes (percentile among expressed genes)	Expression, counts per thousand (percentile)	Detection odds ratio	Expression-matched reference genes	Percentile of focal gene	One-sided empirical p
EBI3	0.198% (23.3rd)	0.0003 (22.6th)	39.13	1,285	99.9th	0.0008
IL27	1.702% (45.4th)	0.0029 (45.0th)	1.23	1,128	69.9th	0.30
IL12A	0.158% (20.3rd)	0.0003 (19.6th)	0.67	1,674	38.4th	0.62
IL23A	0.713% (37.2nd)	0.0012 (36.4th)	0.60	1,155	14.9th	0.85

Principal cohort; 7,164 septic and 2,526 healthy monocytes; 18,865 genes expressed in healthy monocytes. IL27 is detected 8.6-fold more often than EBI3 at baseline and sits higher in the expressed transcriptome on both detection and expression, so the chain showing the strong induction is the less well detected of the two. Reference genes are matched to the focal gene within ±30% on expression in healthy monocytes; the detection-matched construction of Table S7 gives the same percentiles for EBI3 and IL27 (99.9th and 69.9th). Detection odds ratios carry a Haldane 0.5 continuity correction applied identically to the focal gene and to every reference gene, and the inferential quantity is the percentile within the reference distribution rather than the raw odds ratio.

Table S10. Empirical characterisation of the induction magnitude this assay registers at each chain's own detection and expression level.

Reference set matched to	n	OR ≥ 2	OR ≥ 3	OR ≥ 5	OR ≥ 10	Largest reference OR	97.5th percentile
IL27, detection-matched	1,110	71 (6.40%)	27 (2.43%)	9 (0.81%)	3 (0.27%)	19.64 (FAM20A)	2.98
IL27, expression-matched	1,128	74 (6.56%)	29 (2.57%)	8 (0.71%)	2 (0.18%)	18.86 (SLC39A8)	3.01
EBI3, detection-matched	1,297	128 (9.87%)	43 (3.32%)	18 (1.39%)	4 (0.31%)	46.74 (AMPH)	3.58
EBI3, expression-matched	1,285	125 (9.73%)	41 (3.19%)	15 (1.17%)	4 (0.31%)	46.74 (AMPH)	3.28

At IL27's own level the assay registers inductions up to 18.9-fold, and an odds ratio of about 3.0 would clear the matched null; the observed IL27 odds ratio is 1.23. No reference gene at IL27's level reached EBI3's odds ratio of 39.1, so the calibration supports the statement that an induction large enough to clear the matched null would have been registered, and not the stronger statement that an EBI3-magnitude induction would have been detectable at IL27's level. Reference distributions are wider at the lower detection level occupied by EBI3, which is why the percentile within the matched distribution, rather than the raw odds ratio, is used for inference throughout.

S4. Same-cell co-detection and benchmark calibration

Table S11 gives the structure-preserving permutation null and Table S12 the chain-to-chain ratios for every pre-frozen pair under both comparators; the chain-level detection values and the recovery of injected co-detection effects are shown in Figures 4C and 4H. Because injected effects of defined size were recovered by the same pipeline, the observed upper bound of 1.75 excludes a positive association of benchmark magnitude.

Detection-regime calibration placed the IL-27 pair at the centre of 376 matched random gene pairs, while the C1q pairs exceeded every random pair and calprotectin occupied the upper edge of the random-pair range. This separation across the same accessible detection regime reinforces the pair-specific nature of EBI3–IL27 co-detection.

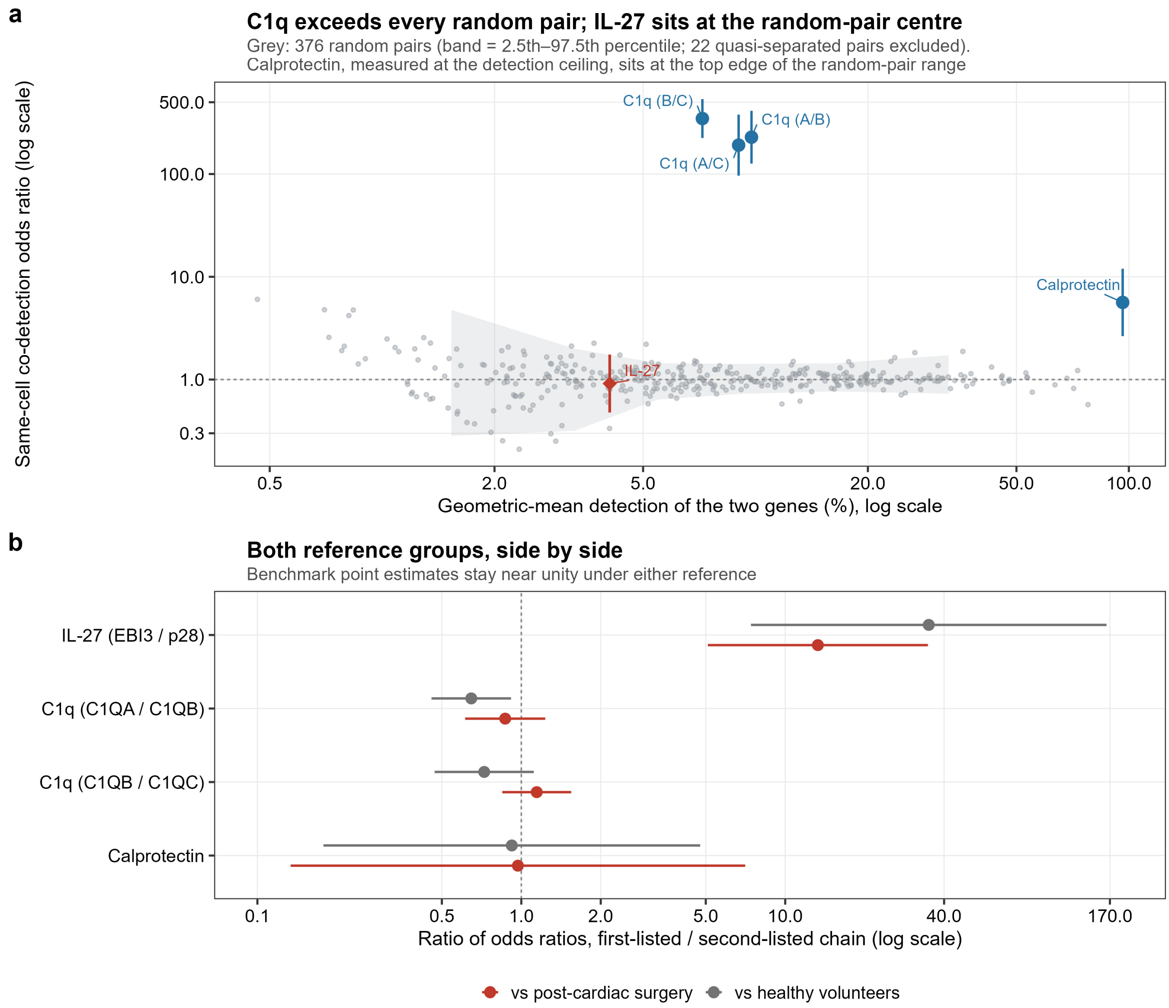

Figure S1. Detection-regime calibration and dual-reference concordance. (a) Same-cell co-detection odds ratio versus geometric-mean detection of the two genes: 376 random gene pairs (grey; band, 2.5th–97.5th percentile; 22 quasi-separated pairs excluded), obligate benchmark complexes (blue) and

IL-27 (red). Error bars, 95% CI from patient-clustered models. (b) Chain-to-chain ratios of odds ratios under both non-infected comparators; benchmark point estimates remain near unity under either reference.

The benchmark set was restricted to obligate complexes detected in the same cells across an overlapping abundance range.

Table S11. Structure-preserving permutation null for same-cell co-detection.

Complex	Observed OR	Permutation null, median	Percentile of the observed value	Empirical *p* (two-sided)
IL-27 (*EBI3* ~ *IL27*)	0.92	1.02	27th	0.71
C1q (*C1QA* ~ *C1QB*)	228.3	6.40	100th	< 0.0005
C1q (*C1QB* ~ *C1QC*)	346.7	6.91	100th	< 0.0005
Calprotectin (*S100A8* ~ *S100A9*)	5.63	3.24	100th	< 0.0005

The structural null differs by pair; observed IL-27 co-detection was near its own null, whereas all three benchmark pairs exceeded every permutation.

Table S12. Chain-to-chain ratios of odds ratios for the pre-frozen benchmark set.

Complex	Contains *EBI3*	vs post-cardiac surgery	vs healthy volunteers
IL-27 (*EBI3*/*IL27*)	yes	13.30 (5.09–34.74)	34.99 (7.42–165.08)
IL-35 (*EBI3*/*IL12A*)	yes	not estimable	61.59 (10.31–367.80)
IL-39 (*EBI3*/*IL23A*)	yes	13.52 (5.07–36.08)	71.75 (18.87–272.80)
C1q (*C1QA*/*C1QB*)	no	0.87 (0.61–1.23)	0.65 (0.46–0.91)
C1q (*C1QB*/*C1QC*)	no	1.14 (0.85–1.55)	0.72 (0.47–1.12)
Calprotectin (*S100A8*/*S100A9*)	no	0.97 (0.13–7.05)	0.92 (0.18–4.76)
CCL2/CCL8	no	not estimable	0.27 (0.04–1.90)
PF4/CXCL8	no	1.09 (0.46–2.57)	1.17 (0.58–2.36)

S5. The EBI3-associated transcriptional state

The 629-gene EBI3-positive signature was calibrated with 30 detection-matched genes. Subset adjustment defined a 99-gene core EBI3-associated state while preserving C1QA/C1QC.

EBI3 detection was 4.54% in classical, 25.24% in intermediate and 18.11% in non-classical monocytes.

Table S13. Differential expression with and without adjustment for protein-defined monocyte subset.

Model	Pseudobulk columns	Patients	Genes tested	FDR < 0.05
Unadjusted (Section 3.3)	24	12	7,234	**629**
Subset as covariate	44	12	4,158	**99**
Classical monocytes only	18	9	5,766	16
Intermediate monocytes only	18	9	3,472	3
Non-classical monocytes only	8	4	3,585	0

Subset adjustment retained a 99-gene EBI3-associated state. Leave-one-patient-out refitting preserved the direction of all 99 genes in every iteration, with genome-wide log2 fold changes highly concordant with the full model (Spearman ρ 0.931 to 0.991). The number of genes at FDR < 0.05 ranged from 55 to 106 (median 93); C1QC remained significant in all twelve refits and C1QA in nine of twelve.

Table S14. Leave-one-patient-out refits of the subset-adjusted EBI3-positive versus EBI3-negative contrast (12 patients; Figure 3H).

Patient removed	Genes at FDR < 0.05	Spearman ρ vs full model	Direction concordance, 99 genes	*C1QA FDR*	*C1QC FDR*
S072	97	0.977	100%	0.0377	0.0035
S076	79	0.974	100%	0.0220	0.0005
S078	105	0.941	100%	0.0777	0.0215
S082	55	0.979	100%	0.0678	0.0086
S091	96	0.990	100%	0.0266	0.0021
S095	106	0.960	100%	0.0350	0.0041
S098	89	0.991	100%	0.0284	0.0026
S101	99	0.948	100%	0.0214	0.0083
S105	80	0.946	100%	0.0275	0.0040
S109	99	0.991	100%	0.0339	0.0043
S113	81	0.931	100%	0.0663	0.0105
S119	69	0.968	100%	0.0219	0.0034

Table S15. External projection of the frozen 99-gene EBI3-associated signature.

Cohort	Platform	Septic samples	Spearman ρ	Percentile against matched random gene sets	Empirical *p*
SCP548	10x 3′	39	+0.431	99.2nd	0.008
SCP3740	10x 5′	52	+0.738	100th	< 0.002
GSE279451	10x 5′, CITE-seq	40	+0.256	91.6th	0.084
**Pooled (DerSimonian-Laird, Fisher z)*		131	**+0.510 (0.148–0.752)**		**0.008**

The frozen 99-gene signature projected positively into all three external cohorts, with Spearman correlations from +0.256 to +0.738. The DerSimonian-Laird pooled estimate was +0.510 (95% CI +0.148 to +0.752; p = 0.008). A REML/Hartung-Knapp sensitivity estimate remained +0.510 (95% CI −0.31 to +0.89), with all three cohorts directionally concordant.

Table S16. The L1 classifier for EBI3 status with MS1-defining genes hard-excluded from the candidate feature set.

Candidate feature set	Features	Cross-validated AUC (95% CI)	Non-zero features	MS1 genes selected
Original: MS1 genes present, selection left to the penalty	10,122	0.904 (0.880–0.928)	19	None of the five-gene definition; CD163 of the extended set
Five-gene MS1 definition hard-excluded	10,117	0.904 (0.880–0.928)	19	None of the five-gene definition; CD163 of the extended set
Twelve-gene extended MS1 set hard-excluded	10,110	0.904 (0.879–0.929)	18	None

All three fits use the same sample-wise fold assignment, the same lambda.1se rule, the same seed and the same circularity screen, which hard-excludes genes correlated with EBI3 at |r| > 0.30 (C1QB, FCGR3A, LYZ). In the original analysis the MS1-defining genes were present in the candidate set and were not selected by the penalty; excluding them outright leaves performance unchanged. CD163 belongs to the broader twelve-gene MS1-associated set but not to the five-gene score used in the main text.

S6. Receiver-side calibration and positive-control validation

Receptor-positive cells defined by both IL27RA and IL6ST showed β = −0.0151 (95% CI −0.0345 to +0.0043), whereas the same analysis recovered a positive IL-15 receptor-linked response, validating sensitivity to a receptor-associated signal.

In 200 detection-matched receptor substitutions, the null 95% coefficient range was −0.0148 to +0.0258; the observed estimate lay at its lower edge.

Table S17. Minimum-detectable-effect calibration of the receiver-side module: effects of known size added to the module score of the dual-receptor-positive cells and passed through the identical patient-clustered model.

Injected effect (module-score units)	As a multiple of the in-vivo IL-15 response	Refitted coefficient	95% confidence interval	Interval excludes zero
0.0000 (observed data)	0.00×	−0.0151	−0.0345 to +0.0043	No
0.0115	0.25×	−0.0035	−0.0229 to +0.0159	No
0.0231	0.50×	+0.0080	−0.0114 to +0.0274	No
0.0300	0.65×	+0.0149	−0.0045 to +0.0343	No
0.0346	0.75×	+0.0196	+0.0002 to +0.0390	Yes
0.0462	1.00×	+0.0311	+0.0117 to +0.0505	Yes
0.0600	1.30×	+0.0449	+0.0255 to +0.0643	Yes

Septic T and NK cells of the principal cohort; the analysis set is the 2,752 cells that are either positive for both receptor chains or negative for both, of which 317 are dual-receptor-positive. A constant of size δ was added to the module score of the dual-positive cells only, and the resulting data were refitted with the same patient-clustered linear model, the same depth covariate and the same t(G−1) reference distribution used for the primary estimate (G = 26 patients); detection was defined in advance as exclusion of zero by the 95% interval. The smallest injected effect meeting that criterion is 0.0346 module-score units, which is 0.75 times the IL-15 response measured in the same cells by the same procedure (+0.0462, 95% CI +0.0377 to +0.0546) and 0.27 times the standard deviation of the module score. IL-15 is used as the reference because it is the only in-vivo effect magnitude available in this dataset; it does not signal through gp130, so it calibrates the sensitivity of the dual-chain stratification rather than the sufficiency of gp130. An IL-27-associated response smaller than 0.0346 units would not have been registered by this analysis and cannot be excluded.

S7. Patient-level and cross-compartment validation

Eight bulk cohorts provided patient-level severity, outcome and cross-compartment analyses, complementing the monocyte-resolved subunit-coordination results.

Table S18. Severity and outcome associations in whole-blood bulk (GSE185263).

Score	SOFA, β (n = 345)	In-hospital death, OR (52/345)	Death AUC (n = 342)

Score	SOFA, β (n = 345)	In-hospital death, OR (52/345)	Death AUC (n = 342)
**EBI3 / p28 ratio**	—	—	0.530 (0.44–0.62)
**EBI3**	−0.008 (p = 0.37)	1.03 (p = 0.91)	0.498 (0.41–0.59)
**MS1**	+0.020 (p = 0.015)	2.11 (1.21–3.70)	0.580 (0.49–0.67)
p28	−0.011 (p = 0.26)	0.74 (p = 0.27)	0.460 (0.38–0.54)

Table S19. Patient-level discrimination within monocytes (GSE216009) across four non-infected comparators.

Score	vs healthy	vs convalescence	vs cardiac surgery	vs all non-infected	Worst comparator
**EBI3 / p28 ratio**	0.872 (0.73–1.00)	0.859 (0.74–0.98)	**0.852 (0.72–0.99)**	0.860 (0.76–0.97)	**0.852**
**EBI3**	0.910 (0.80–1.00)	0.936 (0.85–1.00)	0.769 (0.60–0.93)	0.876 (0.78–0.98)	0.769
**MS1**	0.994 (0.98–1.00)	0.991 (0.97–1.00)	0.654 (0.42–0.89)	0.885 (0.78–0.99)	0.654
p28	0.596 (0.32–0.87)	0.679 (0.48–0.88)	0.374 (0.16–0.59)	0.559 (0.39–0.73)	0.374

Table S20. Cross-compartment discrimination across the cohorts that permit the comparison.

Cohort	Measurand	n (sepsis vs control)	EBI3 AUC (95% CI)	MS1 AUC (95% CI)
GSE216009	$EBI3^{+}$ fraction **within monocytes**	26 vs 7	**0.769 (0.604–0.934)**	0.654 (0.416–0.891)
GSE279448	bulk expression, **PBMC**	132 vs 23	0.653 (0.550–0.757)	0.609 (0.489–0.729)
GSE134347	bulk expression, **whole blood**	156 vs 59	0.553 (0.469–0.637)	**0.804 (0.737–0.870)**
GSE134347	as above, composition-adjusted	156 vs 59	0.477 (0.391–0.562)	0.703 (0.623–0.782)

Table S21. Comparator-invariance calibration against detection-matched null scores.

Score	Floor	Percentile of floor	*p*	Spread	Percentile of stability	*p*	Null pool

Score	Floor	Percentile of floor	*p*	Spread	Percentile of stability	*p*	Null pool
**EBI3 / p28 ratio**	**0.852**	**99.8**	**0.002**	**0.020**	**99.6**	**0.004**	gene pairs
EBI3	0.769	97.8	0.022	0.167	46.8	0.53	single genes
MS1	0.654	94.8	0.052	0.340	19.8	0.80	matched five-gene scores
p28	0.374	—	—	0.306	—	—	—

Comparison	Difference in floor (95%)	Difference in spread (95%)
ratio − EBI3	+0.053 (−0.081 to +0.180)	−0.090 (−0.238 to +0.044)
ratio − MS1	+0.168 (−0.071 to +0.423)	**−0.260 (−0.503 to −0.026)**
ratio − p28 (the chain that is not induced)	+0.451 (+0.192 to +0.690)	**−0.252 (−0.490 to −0.021)**

Table S22. Formal comparison of the EBI3-to-p28 ratio with each subunit alone, within each prespecified comparator arm.

**Comparator arm**	**n (sepsis vs control)**	**AUC ratio**	**AUC EBI3**	**AUC p28**	**Ratio − EBI3 (paired DeLong p)**	**β for p28 given EBI3 (95% CI; LRT p)**
vs healthy volunteers	26 vs 6	0.872	0.910	0.596	−0.038 (0.490)	−0.191 (−1.282 to +0.860; 0.219)
vs cardiac surgery	26 vs 7	0.852	0.769	0.374	+0.082 (0.131)	−0.720 (−2.352 to +0.256; 0.207)
vs sepsis convalescence	26 vs 9	0.859	0.936	0.679	−0.077 (0.197)	−0.169 (−1.231 to +0.851; 0.176)
vs all non-infected	26 vs 22	0.860	0.876	0.559	−0.016 (0.724)	−0.447 (−1.363 to +0.442; 0.131)

All four arms share the same 26 septic patients, so AUCs were compared by the paired DeLong test. The ratio was not significantly more discriminative than EBI3 alone in any arm, and was significantly more discriminative than p28 alone against cardiac surgery (+0.478, p = 0.001) and against all non-infected controls (+0.301, p = 0.009). The final column refits each arm as logit(sepsis) ~ log(EBI3 + 0.1) + log(p28 + 0.1) by Firth penalized likelihood, with profile penalized-likelihood intervals; the ratio is the special case in which the p28 coefficient equals the negative of the EBI3 coefficient. The p28 coefficient did not differ from zero in any arm, and adding p28 did not improve fit. Spearman correlation between the log ratio and EBI3 was +0.849 across all 48 samples. The property that distinguishes the ratio is therefore comparator stability (Table S21 and Figure 5B,E), not discriminative magnitude.

Table S23. Co-occurrence of the EBI3-positive and MS1-high states within and across septic patients.

**Level**	**Quantity**	**Value**

Cell	EBI3-positive and MS1-high	184
Cell	EBI3-positive and MS1-low	379
Cell	EBI3-negative and MS1-high	3,398
Cell	EBI3-negative and MS1-low	3,203
Cell	Double-positive cells expected under independence	281.5
Cell	Observed / expected	0.65
Cell	Odds that an EBI3-positive cell is also MS1-high	0.458
Cell	Patient-clustered linear probability model	β = −0.0574 (95% CI −0.1296 to +0.0148); p = 0.114
Cell	MS1 score, EBI3-positive versus EBI3-negative (median)	−0.287 versus +0.054; $p = 8.8 \times 10^{-25}$
Within patient	Patients with observed / expected below 1	24 of 24 evaluable (median 0.17)
Patient	Patients containing both populations	24 of 26
Patient	Spearman correlation of the two fractions across patients	+0.145; p = 0.480

Septic monocytes of the principal cohort (n = 7,164 cells, 26 patients); MS1-high is the median split of the frozen five-gene score. The two states co-exist within patients rather than defining alternative patient phenotypes: both are present in 24 of 26 patients and their patient-level frequencies are not inversely related. Within a patient an individual cell tends to carry one state or the other, and that direction is uniform across every evaluable patient, but the pooled clustered estimate does not exclude the null, reflecting between-patient heterogeneity in both marginal frequencies. The two patients without a detected EBI3-positive cell contributed 53 and 109 monocytes respectively.

S8. Reproducibility, module definition and dataset roles

Table S24. The fixed 41-gene IL-27-associated receiver module, defined in the single-cell cytokine dictionary [32] as the genes induced by IL-27 in at least 10 of 12 human T and NK subsets, and applied unchanged. The five-gene MS1 comparator score used in the patient-level analyses is the mean of scaled expression of RETN, CLU, PLAC8, ALOX5AP and IL1R2, derived from the MS1 markers of [16].

GBP5, GBP1, ETV7, STAT1, TYMP, GBP4, GBP2, IFI44L, PARP9, IRF1, EPSTI1, TRAFD1, APOL6, STAT2, TAP2, JAK2, DTX3L, GBP1P1, HAPLN3, ALPK1, TRIM21, CD274, PGAP1, XRN1, PARP10, PARP12, WARS1, PARP14, APOL2, CD38, MVP-DT, ATG16L1, IFIT3, IFIT2, MTHFD2, TMEM140, TRIM22, TAP1, TAPBPL, DNAJA1, GIMAP6.

The 41-gene module captures the reproducible IL-27-responsive programme shared across at least 10 of 12 human T/NK subsets in the reference cytokine atlas.

Table S25. Datasets and their prespecified analytical roles.

Dataset	Platform	n	Role

Dataset	Platform	n	Role
GSE216009	BD Rhapsody, whole blood + 30 surface markers	48 samples / 39 individuals	principal cohort: detection models, same-cell co-detection, state analyses, surface protein, receiver-side test, patient-level comparators
SCP548	10x 3'	65 donors	cross-cohort meta-analysis
SCP3740	10x 5'	62 donors	cross-cohort meta-analysis
GSE167363	10x, paired at 0 and 6 hours from sepsis recognition	7 donors	replication of the chain-to-chain ratio; per-sample detection
GSE175453	10x	9 donors	replication of the chain-to-chain ratio (analysed with GSE167363)
GSE279451	droplet, with surface protein	40 donors	additional CITE-seq replication; per-sample detection
GSE151263	10x	7 cases, no controls	supporting cell-count context
GSE85243	RNA-seq, Illumina HiSeq 2000	3 paired donors	human macrophage LPS-preconditioning replication
GSE185433	RNA-seq, Illumina NovaSeq 6000	3 paired donors	human macrophage LPS-preconditioning replication
GSE185263	bulk whole blood	348 septic	severity and mortality association
GSE279448	bulk PBMC	132 vs 23	cross-compartment discrimination
GSE134347	bulk whole blood	156 vs 59	cross-compartment discrimination
GSE87218	ATAC, single donor	1 donor	chromatin accessibility context

Dataset	Platform	n	Role
GSE154918	bulk whole blood	105 samples	composition-adjustment assessment
GSE28750	bulk whole blood	30 samples	composition-adjustment assessment
GSE66099	bulk whole blood	276 samples	composition-adjustment assessment
GSE69528	bulk whole blood	138 samples	composition-adjustment assessment
GSE131761	bulk whole blood	129 samples	composition-adjustment assessment

Table S26. Terminology: hedged terms used throughout the paper, what each denotes and what it deliberately does not assert.

Term as used here	What it denotes	What it does not assert
IL-27-associated immunoreactivity	Signal returned by an immunoassay raised against IL-27 or its p28 subunit	That the signal is assembled EBI3–p28 heterodimer rather than free chain or cross-reactive material
p28-associated axis	The biology that is altered when p28-directed reagents are applied	That those effects are mediated by assembled IL-27
EBI3-associated state	The monocyte transcriptional programme that accompanies EBI3 detection	That the cell produces or secretes IL-27
Detection	At least one unique molecular identifier for that transcript in that cell; no imputation	A quantitative expression level, or the presence of the encoded protein
Same-cell co-detection	Both subunit transcripts detected in the same cell in the same library	Physical assembly of the two chains into a heterodimer
Molecular-attribution gap	The coexistence of functional sensitivity to p28-directed perturbation with limited evidence for coordinated heterodimer production	That IL-27 has no role in sepsis, or that circulating measurements are without value
EBI3-to-p28 ratio	The patient-level ratio of the two detection percentages, (EBI3% + 0.1)/(p28% + 0.1)	A measure of heterodimer abundance; its discriminative properties are given in Table S22

Unhedged forms are used only where the referent is the defined molecule itself, for example when describing recombinant IL-27 added to culture or the obligate heterodimeric structure of the cytokine.

S9. Experimental validation: per-sample and per-mouse values

All values are per-sample or per-mouse measurements; reported experimental p values were calculated from these values.

Table S27. Surface HLA-DR by flow cytometry, percentage of cells in the HLA-DR-high gate. Batch A is the tolerance series; batch B is the pharmacological series.

Batch	Arm	n	Mean ± SD	Individual values
A	Untreated	3	62.43 ± 3.27	60.80, 60.30, 66.20
A	Acute LPS	3	47.07 ± 3.50	51.10, 44.90, 45.20
A	Tolerised	3	4.73 ± 3.39	8.63, 2.48, 3.08
B	Vehicle	3	62.90 ± 2.14	64.60, 63.60, 60.50
B	Recombinant IL-27	3	40.53 ± 1.62	39.50, 42.40, 39.70
B	Tolerised + isotype	3	4.31 ± 2.19	2.67, 3.46, 6.79
B	Tolerised + anti-p28	3	46.23 ± 6.73	42.50, 54.00, 42.20

Table S28. Tumour necrosis factor and IL-27-associated immunoreactivity in the same supernatants, pg/mL, n = 8 per arm. IL-27-associated immunoreactivity was measured in batch A.

Batch	Arm	TNF, mean ± SD	IL-27, mean ± SD
A	Untreated	29.02 ± 14.42	17.03 ± 9.25
A	Acute LPS	559.25 ± 46.98	82.34 ± 21.72
A	Tolerised	82.39 ± 19.63	65.27 ± 14.91
B	Vehicle	556.91 ± 75.18	—
B	Recombinant IL-27	230.11 ± 44.04	—
B	Tolerised + isotype	84.64 ± 24.31	—
B	Tolerised + anti-p28	160.91 ± 41.80	—

Table S29. Survival after caecal ligation and puncture, 20 mice per group, followed to 120 h.

Group	Deaths	Survival at 120 h
Sham	0 / 20	100%
Control IgG	18 / 20	10%
Anti-p28 reagent	11 / 20	45%

Table S30. Peritoneal macrophage composition in a separate cohort at 48 h after surgery.

Group	n	LPM %	SPM %	LPM/SPM	Per-mouse ratios
Sham	4	7.75 ± 1.58	6.23 ± 2.04	1.288 ± 0.200	1.203, 1.048, 1.491, 1.409
Control IgG	3	5.91 ± 1.82	23.33 ± 7.56	0.284 ± 0.141	0.373, 0.121, 0.357

Group	n	LPM %	SPM %	LPM/SPM	Per-mouse ratios
Anti-p28 reagent	4	4.76 ± 1.88	7.04 ± 2.25	0.674 ± 0.132	0.645, 0.813, 0.734, 0.504

Table S31. Lactate dehydrogenase release, optical density, eight wells per arm. Batch A is the tolerance series and batch B the pharmacological series; the two batches were read on separate plates and compared within batch.

Batch	Arm	n	Mean ± SD	Individual values
A	Untreated	8	0.2518 ± 0.0173	0.2481, 0.2597, 0.2410, 0.2524, 0.2276, 0.2359, 0.2718, 0.2777
A	Acute LPS	8	0.2493 ± 0.0093	0.2686, 0.2532, 0.2493, 0.2445, 0.2489, 0.2362, 0.2494, 0.2447
A	Tolerised	8	0.2488 ± 0.0136	0.2789, 0.2533, 0.2500, 0.2485, 0.2457, 0.2364, 0.2402, 0.2375
B	Vehicle	8	0.3697 ± 0.0194	0.4064, 0.3735, 0.3808, 0.3630, 0.3660, 0.3508, 0.3429, 0.3743
B	Recombinant IL-27	8	0.3689 ± 0.0158	0.3637, 0.3871, 0.3734, 0.3586, 0.3556, 0.3441, 0.3823, 0.3861
B	Tolerised + isotype	8	0.3692 ± 0.0207	0.3927, 0.3606, 0.3694, 0.3616, 0.3539, 0.3607, 0.4078, 0.3466
B	Tolerised + anti-p28	8	0.3797 ± 0.0214	0.3659, 0.3846, 0.4101, 0.3819, 0.3940, 0.3379, 0.3890, 0.3741

Lactate dehydrogenase release remained stable across the experimental series; all seven within-batch comparisons gave $p \geq 0.33$, supporting the phenotypic shifts as changes in macrophage state rather than differential membrane damage.

Table S32. Interleukin-6 and interleukin-10 in the batch A supernatants, pg/mL, eight wells per arm. Samples were assayed at a 1:5 dilution and values are reported after correction for that dilution.

Analyte	Arm	Mean ± SD	Individual values
IL-6	Untreated	31.14 ± 21.72	27.49, 19.38, 3.05, 73.25, 14.75, 48.92, 29.81, 32.47
IL-6	Acute LPS	304.22 ± 82.27	495.59, 252.22, 256.12, 292.58, 324.63, 288.17, 288.68, 235.77
IL-6	Tolerised	89.39 ± 27.59	69.05, 76.48, 119.23, 134.36, 86.85, 95.54, 86.43, 47.18

Analyte	Arm	Mean ± SD	Individual values
IL-10	Untreated	80.99 ± 36.75	53.93, 91.87, 71.96, 146.28, 72.27, 76.26, 24.05, 111.27
IL-10	Acute LPS	272.15 ± 41.52	287.27, 211.06, 259.18, 327.42, 316.51, 284.33, 271.86, 219.55
IL-10	Tolerised	534.37 ± 171.17	580.04, 385.70, 361.17, 634.13, 843.36, 652.37, 417.98, 400.16

All acute and tolerised samples were within the calibrated range. Relative to acute LPS, the tolerised arm showed lower IL-6 (Tukey $p = 1.3 \times 10^{-7}$) and higher IL-10 (Tukey $p = 1.5 \times 10^{-4}$); acute LPS induced both cytokines relative to untreated cells (Tukey $p = 2.1 \times 10^{-9}$ and $p = 0.0038$).

Table S33. Cells associated with labelled bacteria, percentage of nucleated cells, eight wells per arm. Each well was scored from two to five randomly selected fields (67 fields and 3,918 cells in total); the well was the prespecified unit of analysis.

Arm	n	Mean ± SD	Per-well values
Untreated	8	69.98 ± 3.26	67.8, 75.4, 69.3, 66.9, 72.6, 66.3, 68.7, 73.0
Acute LPS	8	87.05 ± 2.93	88.5, 85.8, 88.3, 87.5, 90.4, 80.6, 88.3, 87.0
Tolerised	8	50.10 ± 9.09	40.2, 52.4, 62.1, 55.9, 39.5, 39.3, 54.4, 57.1

The bacterial-association assay resolved the expected bidirectional response (ANOVA $p = 1.4 \times 10^{-10}$): acute LPS increased the proportion above untreated cells (Tukey $p = 2.3 \times 10^{-5}$), whereas the tolerised arm fell below both untreated ($p = 2.8 \times 10^{-6}$) and acute LPS ($p = 7.7 \times 10^{-11}$). Pooled positive/total counts within each well yielded closely concordant estimates of 70.2%, 87.8% and 50.4%, confirming robustness to aggregation method.
